# Fundamentals of Thermal Transport and Energy Conversion in Ultra‐High Temperature Ceramics: From Microscale Mechanisms to Macroscopic Properties

**DOI:** 10.1002/advs.202600072

**Published:** 2026-07-27

**Authors:** Zhipeng Pei, Yue Li, Jiayu Xing, Yue Zhu, Ru Zhang, Huan Wu, Zihao Qin, Gang Pei, Man Li

**Affiliations:** ^1^ Department of Thermal Science and Energy Engineering School of Engineering Science University of Science and Technology of China Hefei Anhui P. R. China; ^2^ School for Engineering of Matter Transport & Energy Arizona State University Tempe Arizona USA; ^3^ Department of Mechanical and Aerospace Engineering University of California Los Angeles California USA

**Keywords:** electron‐phonon interaction, thermal radiation and thermoelectrics, thermal transport, ultra‐high temperature ceramics

## Abstract

Ultra‐high temperature ceramics (UHTCs) are pivotal materials for applications in extreme environments, such as hypersonic flight, advanced nuclear systems, and next‐generation energy conversion, where their thermal transport and radiative properties dictate performance. This review examines the thermal transport and energy conversion fundamentals of UHTCs. It covers the unique characteristics and extreme‐condition stability of these materials, alongside the governing heat transfer mechanisms—including phonon‐phonon, electron‐phonon, and photon‐matter interactions. The influence of micro/nanostructure and composition on thermal conductivity and high‐temperature radiative properties is analyzed. The review also assesses the potential of UHTCs for thermoelectric energy conversion and highlights the growing role of physics‐informed machine learning in accelerating their discovery and optimization, from high‐throughput screening to multiscale modeling. Key challenges and future opportunities in predictive design, scalable synthesis, and microstructure control for extreme‐environment applications are critically assessed, offering a roadmap for advancing UHTCs toward future technological demands.

## Graphical abstract

This review critically examines thermal transport and radiative properties of ultra‐high temperature ceramics for hypersonic flight, advanced nuclear systems, and next‐generation energy conversion devices. It explores phonon–photon–electron interactions, microstructural engineering, thermoelectric conversion, and machine learning‐accelerated multiscale discovery. Key challenges and future predictive design roadmaps for extreme environments are comprehensively assessed.

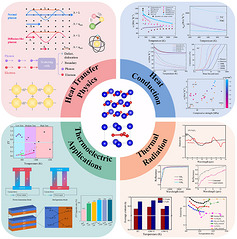

## 1 Introduction

**FIGURE 1:**
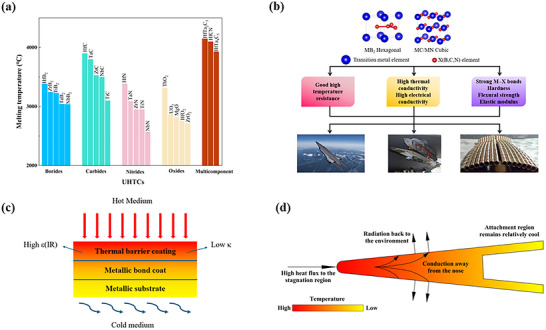
Characteristics and Applications of Ultra‐high temperature ceramics (UHTCs). (a) Melting points of high‐temperature materials [1, 6, 7, 8, 9, 10]. (b) Crystal structure, physical properties, and applications of UHTCs [6, 18, 19]. The first image is reproduced under the terms of the CC BY 4.0 license [19]. Copyright Lu et al. The second image is in the public domain from NASA [6]. The third image is reproduced with permission [18]. Copyright 2010, Wiley. (c) Schematic of a traditional thermal boundary coating design. (d) UHTC leading‐edge thermal management concept. Energy is transferred into the interior through thermal conduction and transmitted back to the environment by radiation.

Materials capable of operating in extreme environments form the foundation of next‐generation energy, space, and hypersonic technologies [1, 2, 3]. For instance, realizing human aspirations for nuclear fusion power requires materials that interact with the core temperatures exceeding 100 million °C, demanding exceptional thermal stability, radiation resistance, and tolerance to corrosive environments [4, 5]. Even within conventional energy systems, the second law of thermodynamics dictates that higher‐temperature heat sources significantly improve energy conversion efficiency, necessitating the development of advanced high‐temperature materials. Similarly, aerospace vehicles during launch and atmospheric re‐entry, as well as hypersonic vehicles in sustained flight, require materials that maintain structural integrity at extreme temperatures. Ultra‐high temperature ceramics (UHTCs) have emerged as a key material family addressing these challenges. As shown in Figure 1a, UHTCs are advanced materials that possess high melting temperatures; they could also maintain high mechanical strength, oxidation resistance, as well as exceptional thermal, electrical conductivity and emissivity at temperatures above 2000°C [1, 6, 7, 8, 9, 10]. These properties enable UHTCs to operate reliably under severe thermal and mechanical stresses, ensuring long operational life and stable performance in critical aerospace, defense, and energy applications. As shown in Figure 1b–d, their typical structures and performance bring many applications, including thermal barrier coating and thermal protection systems (TPS) for hypersonic flight and atmospheric re‐entry, nuclear power system components, rocket propulsion components, and space exploration structures.

Beyond structural and thermal applications, UHTCs also demonstrate significant potential for high‐temperature thermoelectric energy conversion. Recent experimental observations and theoretical predictions indicate promising thermoelectric coefficients in some UHTC compositions [11, 12]. Thermoelectric generators are critically important for reliable power generation in extreme environments inaccessible to human maintenance, such as deep space or the ocean depths [13, 14, 15]. However, conventional thermoelectric materials such as Bi_2_Te_3_ and PbTe are difficult to meet the extreme environmental demands due to resource scarcity, decomposition temperatures below ∼900 K, and insufficient stability under extreme conditions [16, 17]. Additionally, increasing the operating temperature of the hot ends typically improves thermoelectric conversion efficiency. The high melting point and oxidation resistance of UHTCs provide possibilities for their application in high‐temperature and high‐efficiency thermoelectric conversion.

UHTCs are typically composed of transition metal borides, carbides, nitrides, and oxides [20, 21, 22]. Table 1 summarizes the key properties of representative UHTCs, largely derived from extensive trial‐and‐error experimentation [2, 5, 21, 23, 24, 25, 26, 27, 28, 29, 30, 31, 32, 33, 34]. However, due to variations in synthesis methods, many physical properties exhibit significant uncertainty. For example, even extensively studied ZrB_2_ and HfB_2_ systems – favored for their high melting points and chemical inertness – display a wide range of experimentally reported thermal conductivity values (e.g., 60–140 Wm^−1^K^−1^ [35, 36, 37]) attributable to different processing routes [38]. Over recent decades, advances in ab initio modeling have enabled accurate prediction of phonon and electron transport behaviors. Concurrently, ab initio studies revealing unique electronic structures have driven interest in boron carbide B_4_C and nitrides like ScN for high‐temperature thermoelectric applications [39, 40, 41, 42]. The thermal radiative properties of UHTCs are equally critical in high‐temperature systems where radiative thermal resistance becomes comparable to conductive resistance, for example, in radiative thermal protection systems (Figure 1c,d). In such scenarios, the low thermal conductivity of UHTCs provides effective insulation by impeding heat penetration from the external environment, while thermal radiation enables the generated energy to be dissipated from the surface, thereby keeping the protected devices relatively cool. Despite their inseparable role in the overall heat transfer process, conduction and radiation originate from fundamentally different physical mechanisms—the former arises from diffusive transport of phonons and electrons, while the latter involves electromagnetic wave propagation. Recent studies demonstrate that emissivity and reflectance can be selectively tuned across target wavelength ranges through methods like doping and microstructure engineering, enabling precise control of photonic energy absorption/emission. For example, ZrB_2_‐SiC exhibits the highest IR emissivity of 0.94 at 750°C with 10% Pr_6_O_11_ doping [43] and as these property manipulation techniques advance, UHTCs with tailored spectral responses will become increasingly vital. More recently, physics‐informed machine learning (ML) has emerged as a powerful tool for high‐throughput screening of novel UHTC compositions. Multicomponent systems were discovered to possess the highest known melting points (e.g., HfCN at ∼4300 K and HfTaC at ∼4420 K [8, 9, 10]) by ML‐driven screening of UHTCs.

**TABLE 1:** Basic properties of various ultra‐high temperature ceramics [2, 5, 12, 21, 31, 32, 33, 34, 44, 45, 46, 47, 48].

Material	Melting point (°C)	Density (g/cm^3^)	CTE (10^−6^/k)	Thermal conductivity (Wm^−1^K^−1^)	Electrical resistivity (µΩ·cm)	Seebeck coefficient (µV/K)	Emissivity	Elastic modulus (GPa)	Hardness (GPa)	Fracture Toughness (MP·am^0.5^)
ZrB_2_	3245	6.1	5.5–8.3	60‐140	10–32		0.35–0.45	340–500	20–25	1.9
HfB_2_	3380	11.2	6.3–7.6	90–120	8.8–11		0.40–0.45	480	21–28	3.1
TiB_2_	3225	4.5	7.6–8.6	60–80	16–28	3‐8		500–560	24	4–5
TaB_2_	3040	12.5	8.2–8.8		33		0.4	551	20–25	4–5
NbB_2_	3036	6.9				2		637	21	4
ZrC	3530	6.6	6.82	10‐30	68		0.9	480	16.4	3.1
HfC	3900	12.8	6.6	15–25	109		0.9	300–340	18.5	3.4
TiC	3100	4.9	7.5–7.7	32	52.5	5–10	0.6–0.7	451	30	
TaC	3800	14.5	6.6–8.4	20–40	30–42	2	0.43	470–540	14–19	3.4
NbC	3500	7.6	6.7	15	74		0.79	338	20	
ZrN	2950	7.3	7.2	20	20.5	1.5		380	16.6	1.8
HfN	3385	13.9	6.9	93	33			420	16	1.9
TiN	2950	5.4	9.3	63	21.7		0.26	463	18.6	2
TaN	3090	13.4	3.2	3–8, 1100 (θ phase)	128–135	7–14		490	10.8	1
HfCN	4100	12.758		20	47–67				19	6.7

**FIGURE 2:**
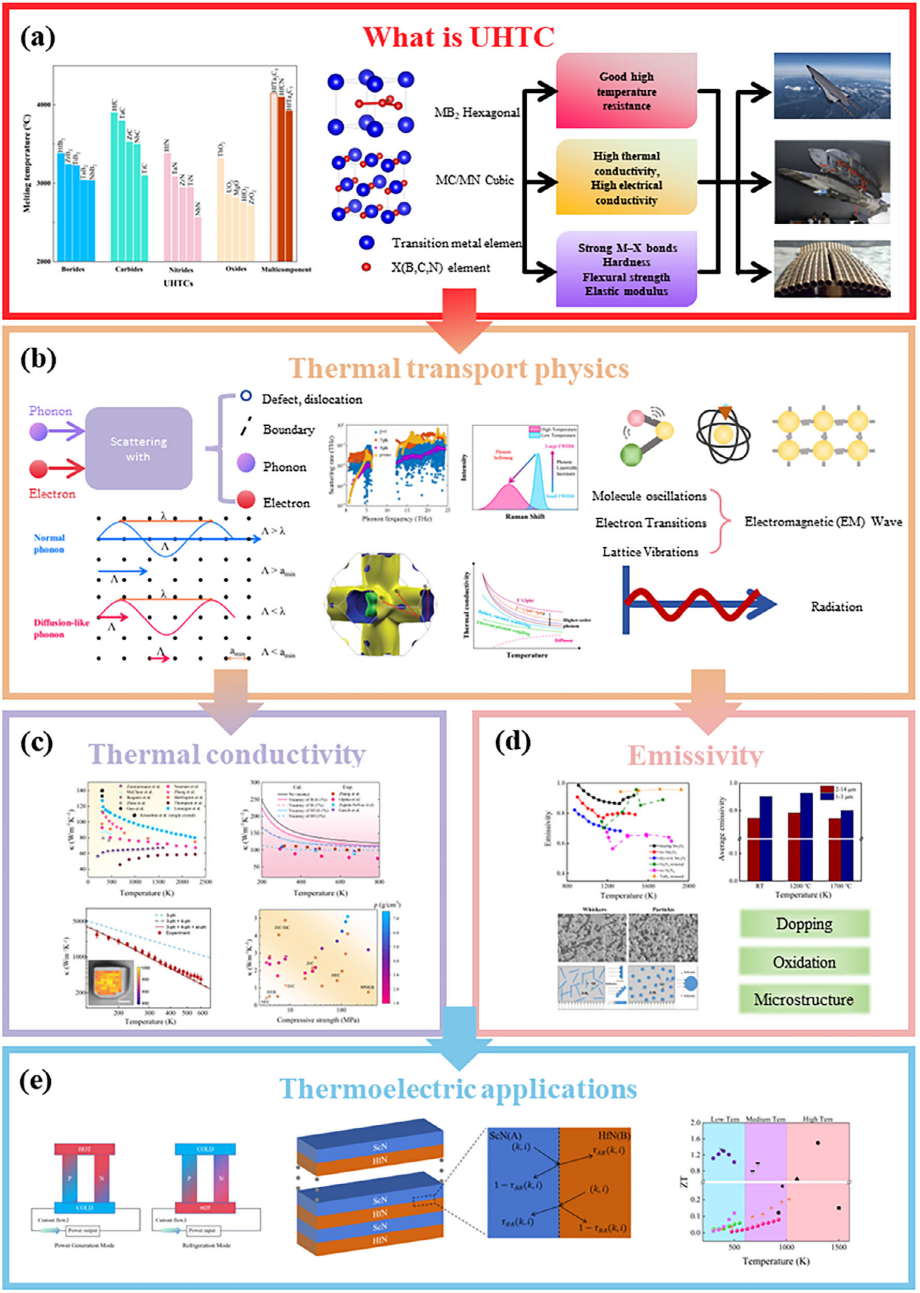
The framework of this article is as follows: it begins with an introduction to UHTCs (a). The first image is reproduced under the terms of the CC BY 4.0 license [19]. Copyright Lu et al. The second image is in the public domain from NASA. The third image is reproduced with permission [18]. Copyright 2010, Wiley. This is followed by an in‐depth discussion of heat transfer mechanisms (b). Reproduced with permission [49]. Copyright 2023, Youke Publishing. Reproduced with permission [50]. Copyright 2018, APS. The discussion then moves to thermal conductivity (c). Reproduced with permission [51]. Copyright 2026, AAAS. Radiative properties (d) are examined next. Reproduced with permission [52]. Copyright 2015, Elsevier. Finally, thermoelectric applications (e) are addressed.

As shown in Figure 2, this review systematically examines the thermal and thermoelectric properties of UHTCs from fundamental mechanisms to applications and advanced design strategies. First, we establish the theoretical foundation, dissecting the atomic‐scale phonon‐phonon and electron‐phonon interactions governing high‐temperature heat and charge transport. The fundamental laws of thermal radiation are introduced in detail. Building on this basis, we rigorously evaluate the thermal conductivity of key UHTC systems (borides, carbides, nitrides), and briefly introduce machine learning as a complementary approach for predicting thermal conductivity, before explicitly linking these properties to micro/nanostructural engineering approaches and highlighting the significant property variations arising from diverse synthesis routes. Then, based on the controllable radiative properties of UHTCs, we introduce the applications of material thermal radiation and strategies for modifying its properties, which are related to the composites, roughness, surface microstructure, and so on. Finally, we evaluated the thermoelectric properties of UHTCs to explore their potential for thermoelectric energy conversion in extreme environments. By correlating thermal transport mechanisms with electronic transport characteristics, we systematically analyzed the key performance indicators and optimization pathways for these materials in thermoelectric applications.

## 2 Heat Transfer Physics at High Temperature

Heat transfer is one of the most universal physical phenomena in nature and engineering, describing the process of thermal energy transfer from high‐temperature regions to low‐temperature regions due to temperature differences. According to the second law of thermodynamics, as long as a temperature gradient exists, heat transfer occurs spontaneously until the system reaches thermal equilibrium. For UHTCs used in extreme environments, a profound understanding of heat transfer mechanisms is not only the theoretical foundation for evaluating their thermal protection performance but also the key to guiding the microstructural design and property optimization of these materials.

**FIGURE 3:**
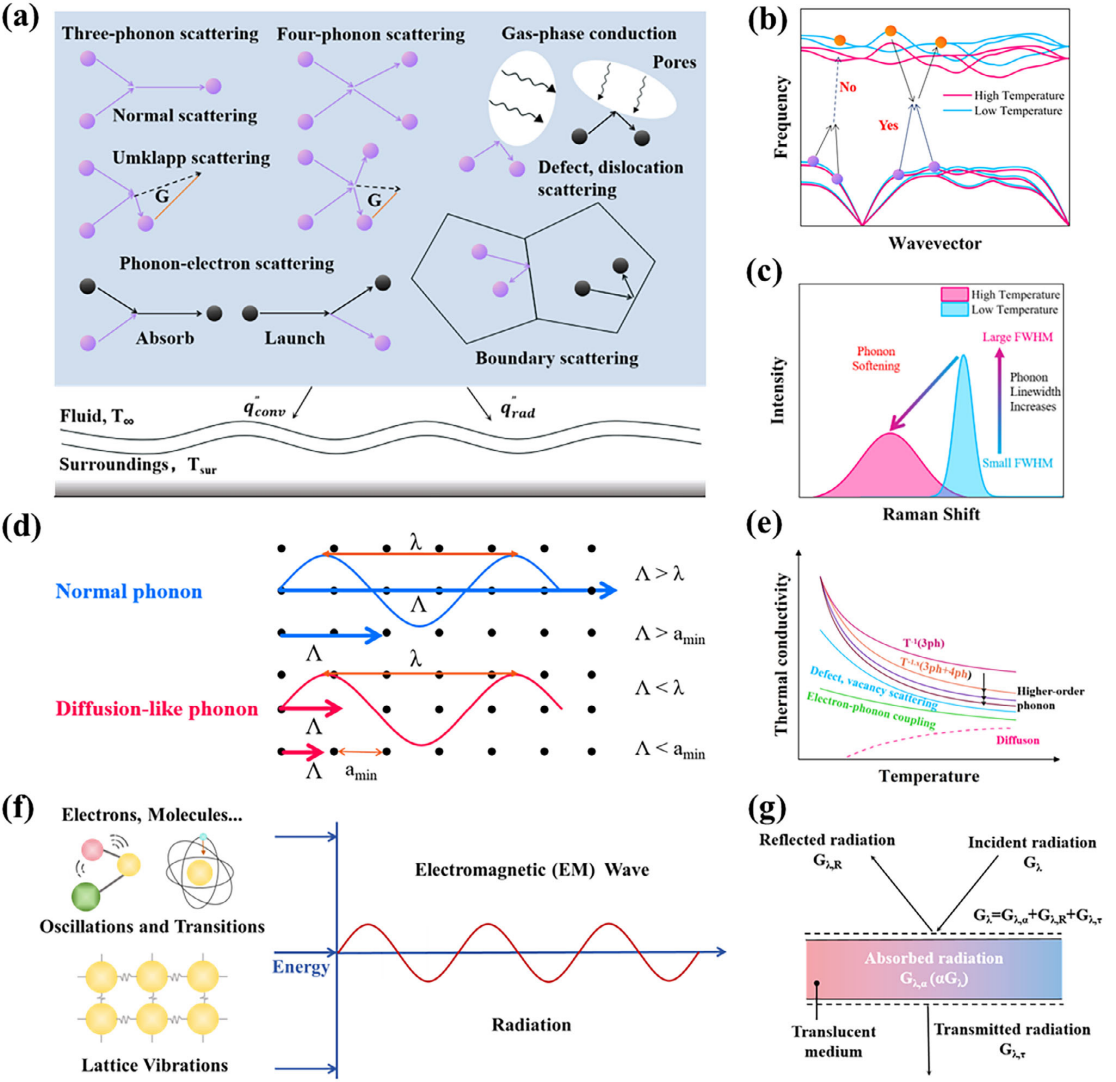
Heat transfer mechanisms. (a) The energy transfer decomposition framework encompasses three fundamental modes of heat transfer: conduction, convection, and radiation. Heat conduction primarily relies on electrons and phonons as energy carriers, which undergo scattering during transport. Furthermore, due to the presence of pores and grain boundaries within the material, heat conduction can be further subdivided into solid‑phase conduction, interfacial heat transfer, and gas‑phase conduction. The purple represents phonons, and the black represents electrons. (b) A large bandgap suppresses three‐phonon scattering. As the temperature increases, phonons will exhibit significant softening, and the bandgap will narrow, thereby affecting thermal conductivity. (c) Raman diagram of the phonon softening process. The phonon linewidth increases with temperature, indicating intensified phonon scattering. (d) When the mean free path of phonons decreases to be smaller than their wavelength or the minimum lattice spacing of the crystal at high temperatures, a transition from phonons to diffusons occurs. (e) Thermal transport is influenced by various scattering processes. (f) Schematic diagram of the microscopic mechanism of thermal radiation. (g)The light behavior when incident on a material surface and Kirchhoff's law.

From a physical perspective, the microscopic carriers of heat transfer are primarily electrons, phonons (lattice vibration quanta), and photons (electromagnetic waves). As illustrated in Figure 3a, based on the state of the macroscopic medium involved in the heat transfer process, heat transfer is traditionally classified into three fundamental modes: heat conduction, heat convection, and thermal radiation. Among these, heat conduction can be further subdivided into solid‑phase conduction, interfacial heat transfer, and gas‑phase conduction.

As the fundamental way of thermal energy transport within solid, heat conduction is described by Fourier's Law, which states that the rate of heat transfer is proportional to the temperature gradient and opposite to the direction of the temperature gradient:

(1) j=−κ∇T

where *j*, κ and *T* are heat flux density, thermal conductivity, and temperature. Thermal conductivity is a key metric defining the material's capability to conduct heat. In crystals, thermal transport is dominated by phonons and electrons. Therefore, thermal conductivity can be divided into phonon thermal conductivity, electronic thermal conductivity. In most UHTCs, the contributions of both are non‐negligible, and show drastic temperature dependence.

Thermal radiation heat of a surface can be calculated with Stefan–Boltzmann's law and the radiative flux scales with the fourth power of temperature:

(2) $$E=\varepsilon \sigma T^{4}$$

here, σ is the Stefan–Boltzmann constant and ε is the emissivity of a surface. Different from the diffusive processes of heat conduction, radiation heat transfers in the form of electromagnetic waves.

In the operating environment of the UHTC materials, conduction governs the internal heat transport within the material, while radiative heat transfer dictates the cooling or thermal insulation at the surface. Thus, understanding both the conduction and radiation properties is essential for a comprehensive assessment of the material. Of course, convection and phase‐change heat transfer also play a role in the practical applications of UHTCs. In extreme service environments such as hypersonic flight, UHTCs serve as critical materials for TPS and are subjected to severe aerodynamic heating on their surfaces. In this context, convective cooling, through the circulation of coolants, can effectively remove absorbed heat and achieve a balance between heat increment and loss, thereby maintaining the structural temperature within acceptable limits [53, 54]. Furthermore, phase‐change cooling, which utilizes coolants injected through porous UHTC materials into the boundary layer to absorb heat via phase change, has emerged as a key direction for active thermal protection [55]. The scope of this paper is limited to the intrinsic physical properties of UHTCs, specifically internal energy transport via diffusive conduction (electron‐ and phonon‐mediated) and radiative transfer at high temperatures. Accordingly, heat transfer mechanisms involving macroscopic fluid flow and phase change are not covered; readers are directed to other reviews on these topics [56, 57, 58, 59]. The following sections will introduce the fundamental physical mechanisms underlying these two heat transfer modes.

### 2.1 Phonon Thermal Conduction

Phonons are quantized vibrational waves with frequency ω, group velocity υ, and wavevector q, following the Bose–Einstein distribution under equilibrium condition [60]:

(3) $$f_{0}\left(\omega ,T\right)=\frac{1}{\exp\left[\hbar \omega /k_{B}T\right]-1}\;$$

where *k_B_* is the Boltzmann constant. The transport behavior of phonons can be described by the Boltzmann transport equation (BTE):

(4) $$\frac{\partial f}{\partial t}+\upsilon \cdot \nabla f={\left(\frac{\partial f}{\partial t}\right)}_{coll}\;$$

where the left side is the drift term (describing the advection of phonons), and the right side is the collision term (describing the distribution relaxation caused by phonon scattering). By solving the BTE, the phonon thermal conductivity can be calculated from the phonon properties:

(5) $$\kappa^{\alpha \beta }=\sum_{q,p}C_{q,p}v_{q,p}^{\alpha }\Lambda_{q,p}^{\beta }\;$$

where α and β denote the crystal directions and label a phonon mode with wave vector q and polarization p. *C*_*q*, *p*_and $v_{q,p}^{\alpha }$ represent the volumetric specific heat and the group velocity along α direction, respectively. $\Lambda_{\lambda }^{\beta }$ is the effective phonon mean free path (MFP) along β direction. Phonon thermal transport is determined by the phonon dispersion relation and scattering processes. Phonon dispersion describes the relation between phonon frequency and phonon wavevector, which can usually be obtained by neutron scattering experiments or the solution of the lattice dynamical equation. However, it is much more complicated to extract phonon scattering information. As shown in Figure 3a, phonons can scatter with other phonons, defects, boundaries, and electrons. When impurities are present in the crystal, phonons can scatter from sites such as vacancies, isotopes, or other imperfections, known as phonon‐defect scattering. Phonon‐boundary scattering refers to the interaction of a travelling phonon with a material surface, interface, or grain boundary. As shown in Figure 3e, these scatterings reduce the thermal conductivity and are typically temperature‐independent. Phonon‐phonon scattering can be further classified into Normal scattering and Umklapp scattering. In the Normal process, phonon momentum is strictly conserved. However, in the Umklapp process, one phonon wavevector is shifted by a reciprocal lattice vector to fold it back into the first Brillouin zone, ensuring that all wavevectors involved in the scattering remain within the first Brillouin zone. The folding during the Umklapp process destroys the crystal momentum and generates inherent thermal resistance [61, 62].

At low temperatures, the harmonic approximation can well describe lattice vibrations and phonon behavior, since atoms oscillate with small amplitudes and a harmonic expansion of the interatomic potential is sufficient. However, as the temperature rises, the atomic vibrational amplitude increases, and higher‐order anharmonic terms become significant in determining the phonon behavior. The most direct manifestation of anharmonicity is phonon softening: high temperatures induce lattice thermal expansion, which increases the interatomic bond length and weakens the bond strength, thereby causing a red shift of phonon frequency with increasing temperature (the optical branches exhibit a more pronounced red shift than the acoustic branches, as shown in Figure 3b). Phonon softening can usually be directly measured by Raman spectroscopy. As illustrated in Figure 3c, with the increase of temperature, the characteristic peaks in the Raman spectrum shift toward lower wavenumbers (leftward), and the full width at half maximum (FWHM) increases. The Grüneisen parameter (γ) is a key dimensionless physical quantity quantifying the strength of anharmonic effects, defined as the relative rate of change of phonon frequency with crystal volume. A large positive Grüneisen parameter indicates strong anharmonicity in the material and more significant temperature‐induced phonon softening, which usually results in low thermal conductivity.

In addition, according to the Bose‐Einstein distribution, the number of high‐energy phonons increases dramatically as temperature rises, making the four‐phonon scattering process involving four phonons non‐negligible. For decades, the three‐phonon scattering process has been considered to dominate thermal transport in solids, while higher‐order four‐phonon scattering is often overlooked [63, 64]. However, in many materials with a large phonon bandgap, the contribution of four‐phonon scattering can be comparable to or even more prominent than that of three‐phonon scattering. For example, in boron arsenide, a novel III–V semiconductor, the four‐phonon scattering is non‐negligible, and the consideration of four‐phonon scattering reduces the predicted room‐temperature thermal conductivity from 2200 to 1400 Wm^−1^K^−1^ [65]. As shown in Figure 3e, high‐order phonon scattering leads to a significant decrease in the thermal conductivity of materials at high temperatures. Owing to their large atomic mass ratios, strong chemical bonds, and simple lattice structures (e.g., a primitive cell with fewer atoms), UHTCs exhibit a more pronounced separation between acoustic and optical phonon branches in their phonon spectra, forming a characteristic “large phonon band‐gap” (Figure 3b). This large bandgap strongly suppresses three‐phonon scattering, because the combined energy of two acoustic phonons is typically insufficient to bridge the gap and satisfy momentum and energy conservation with an optical phonon. In contrast, four‐phonon scattering can occur through exchanges between two acoustic and two optical modes. At elevated temperatures, the population of high‐energy modes increases, enhancing four‐phonon and even higher‐order anharmonic scattering. Four‐phonon scattering thus plays a key role in the thermal transport of UHTCs, and even higher‐order processes may also become non‐negligible under extreme conditions.

In complex crystals and disordered systems [66], propagating lattice waves contribute less to thermal transport due to the loss of coherence. Instead, the tunneling between vibrational modes makes a larger contribution, giving rise to the so‐called diffuson thermal conductivity (κ_dif_). The thermal conductivity contributed by wave‐like tunneling processes depends on the overlap between phonon energy bands. Simoncelli et al. [67, 68] introduced the Wigner formulation, which provided an expression for κ_dif_:

(6) $$\begin{array}{ccc} K_{\text{dif}} & = & \frac{\hbar^{2}}{k_{B}T^{2}}\frac{1}{VN_{c}}\sum_{q}\sum_{s\neq s^{\prime }}\frac{\omega {\left(q\right)}_{s}+\omega {\left(q\right)}_{s^{\prime }}}{2}V^{\alpha }{\left(q\right)}_{s,s^{\prime }}V^{\beta }{\left(q\right)}_{s^{\prime },s}\\ & & \times \;\frac{\omega {\left(q\right)}_{s}f_{0}{\left(q\right)}_{s}\left[f_{0}{\left(q\right)}_{s}+1\right]+\omega {\left(q\right)}_{s^{\prime }}f_{0}{\left(q\right)}_{s^{\prime }}\left[f_{0}{\left(q\right)}_{s^{\prime }}+1\right]}{4{\left[\omega {\left(q\right)}_{s}-\omega {\left(q\right)}_{s^{\prime }}\right]}^{2}+{\left[\Gamma {\left(q\right)}_{s}+\Gamma {\left(q\right)}_{s^{\prime }}\right]}^{2}}\\ & & \times \;\left[\Gamma {\left(q\right)}_{s}+\Gamma {\left(q\right)}_{s^{\prime }}\right] \end{array}$$

where 𝒱 stands for the volume of the crystal's unit cell, *N_c_* is the total number of unit cells in the crystalline system under consideration, *s*, *s*′ are the phonon branch indices (with $s\neq s^{\prime }$ indicating different phonon modes involved in coherent coupling), *f*_0_(*q*)_*s*_ refers to the equilibrium Bose‐Einstein distribution function for phonons, describing the average occupation number of the *s*‐th phonon mode at thermal equilibrium; *V* ^α^(*q*)_*s*,*s*′_ is the off‐diagonal element of the velocity operator in the phonon representation, and Γ(*q*)_*s*_ denotes the linewidth of the *s*‐th phonon branch at wave vector *q*. Due to enhanced linewidth, wave‐like tunneling becomes more important at high temperatures, driving a fundamental transition in thermal transport mechanisms and ultimately exhibiting weak or even increasing temperature dependence of thermal conductivity. With increasing temperature, phonon transport undergoes a propagative‐to‐nonpropagative conversion: increasing temperature significantly shortens the phonon relaxation time, leading to a substantial reduction in the phonon mean free path (Λ). As shown in Figure 3d, when Λ is smaller than the phonon's own wavelength λ, the phonon cannot fully exhibit wave characteristics; alternatively, when Λ is smaller than the minimum lattice spacing *a_min_* of the crystal, the phonon fails to sample the periodic structure of the lattice, resulting in localized transport behavior. When either condition is satisfied, phonon transport transitions from the propagation of lattice waves to the diffusion of atomic vibrational energy. Under this mechanism, thermal energy transfer no longer relies on the directional movement and collision of phonons, but rather resembles the tunnelling of localized vibrational modes [67, 69].

### 2.2 Electronic Thermal Conduction

Electronic thermal conductivity (κ_e_) dominates heat transport in metals and in semiconductors with high carrier concentration. UHTCs usually possess high κ_e_ [36, 49, 70], due to their high electrical conductivity. Based on Fermi–Dirac statistics, electrons in metals must satisfy the Pauli exclusion principle: only electrons within the energy range near the Fermi surface (approximately k_B_T) can participate in heat transport, since they have available unoccupied states for scattering, enabling them to transport heat. These electrons carry heat by transporting energy relative to the Fermi surface, while scattering processes with phonons, impurities, or other electrons (Figure 3a) limit their mean free paths and thereby κ_e_. In contrast, electrons in fully occupied deep states make no contribution because no empty states are available for them to transit into. The distribution function that measures the number of electrons in the state **k** is $f_{\vec{k}}$. In equilibrium, the distribution function is $f_{\vec{k}}^{0}$, given by:

(7) $$f_{\vec{k}}^{0}=\frac{1}{\exp\left(\frac{E_{\vec{k}}-E_{F}}{k_{B}T}\right)+1}\;$$

where E_F_ is Fermi energy. At the level of transport equations, the BTE is the core tool for describing electronic thermal conduction. Combining the steady‐state solution of the BTE and linearization approximation, the core formula for κ_e_ is derived [71]:

(8) $$K_{e}=\frac{1}{T}\;\left(K_{2}-\frac{K_{1}^{2}}{K_{0}}\right)$$

where

(9) $$K_{n}=-\frac{1}{3}\int {\left({\vec{v}}_{\vec{k}}\right)}^{2}\tau \left(k\right){\left(E_{\vec{k}}-E_{F}\right)}^{n}\frac{\partial f_{\vec{k}}^{0}}{\partial E_{\vec{k}}}d\vec{k}$$

corresponding to electron concentration (K_0_), energy transport (K_1_), and contributions from high‐energy electrons (K_2_) respectively, ${\vec{v}}_{\vec{k}}$, τ(*k*), and $E_{\vec{k}}$ are the electron velocity, the relaxation time, and the electron energy, respectively.

For degenerate systems such as metals, $\frac{\partial f_{0}}{\partial \varepsilon }$ is approximately a delta function at the Fermi surface, so the transport integrals can be simplified to integrals over the Fermi surface. Scattering mechanisms are key to regulating electronic thermal conductivity. During transport, electrons scatter with lattice vibrations (phonons), impurities, defects, and other electrons. According to Matthiessen's rule, the reciprocal of the total relaxation time is the sum of the reciprocals of the relaxation times of individual scattering mechanisms, i.e., $\frac{1}{\tau_{total}}=\sum \frac{1}{\tau_{i}}$, which provides a simplified method for analyzing κ_e_ when multiple scattering mechanisms coexist. Furthermore, the Wiedemann–Franz Law is an important corollary of electronic thermal conduction theory, which establishes the relationship between κ_e_ and electrical conductivity σ: Under the relaxation time approximation, if the relaxation time is same for different electron states on Fermi surface, κ_*e*_ = *L*_0_ σ*T*, where $L_{0}=\frac{\pi^{2}}{3}\;\frac{k_{B}^{2}}{e^{2}}\approx 2.45\times 10^{-8}\;V^{2}/K^{2}$ is the Lorenz constant. In a degenerate metal where the same quasiparticles carry charge and heat simultaneously, and the relaxation time is nearly energy‐independent around the Fermi level, the relaxation time τ cancels between the electrical and thermal conductivities, giving the universal Lorenz number *L*_0_. For degenerate metals, the conditions of the Wiedemann–Franz law are usually satisfied under impurity scattering or at high temperatures dominated by phonon scattering, since the scattering time is nearly energy‐independent around the Fermi level. However, this law can fail in several situations. At intermediate temperatures near the Debye temperature, electron–phonon scattering acquires a strong energy dependence, leading to deviations in the Lorenz number. In non‐degenerate semiconductors or systems with bipolar conduction, the assumption of a sharp Fermi surface breaks down, so the Wiedemann‐Franz relation is no longer applicable. In addition, multiband materials, anisotropic band structures, or hydrodynamic effects can further drive substantial deviations.

### 2.3 Electron‐Phonon Interactions

The electron‐phonon interaction (EPI) is one of the core mechanisms for the thermal and electronic transport behavior of UHTCs (such as transition metal carbides VC/NbC, borides ZrB_2_/TiB_2_, nitrides TiN/HfN, etc.). Its essence is the energy exchange process between electrons and phonons in a crystal. It can be conceptualized as the process in which electrons traverse the lattice's periodic potential field, encountering perturbations that transfer energy and momentum to them, often accompanied by phonon emission or absorption. Phonon is thus influenced by their interactions with electrons, which directly determine κ_ph_ and κ_e_, and the phonon dynamic stability of materials. The physical essence of EPI can be described by the Hamiltonian decomposition [72]:

(10) $$\begin{array}{ccc} \hat{H} & = & {\hat{H}}_{e}+{\hat{H}}_{p}+{\hat{H}}_{ep}\\ & = & \sum_{nk}\varepsilon_{nk}{\hat{c}}_{nk}^{†}{\hat{c}}_{nk}+\sum_{q\nu }\hbar \omega_{q\nu }\left({\hat{a}}_{q\nu }^{†}{\hat{a}}_{q\nu }+\frac{1}{2}\right)\\ & & +\;N_{p}^{-\frac{1}{2}}\sum_{k,q,mn\nu }g_{mn\nu }\left(k,q\right){\hat{c}}_{mk+q}^{†}{\hat{c}}_{nk}\left({\hat{a}}_{q\nu }+{\hat{a}}_{-q\nu }^{†}\right) \end{array}$$

where ${\hat{H}}_{e}$ is the electron Hamiltonian (describing the motion of electrons in the periodic lattice), ${\hat{H}}_{p}$ is the phonon Hamiltonian (describing the quantized mode of lattice vibrations), and ${\hat{H}}_{ep}$ is the electron‐phonon coupling term, ε_*n*k_ is the energy of an electron at wavevector k in band index n, ℏω_qν_ is the energy of lattice vibration at wavevector q in phonon branch index ν, and *N_p_* is the number of unit cells under Bornvon Karman boundary condition. ${\hat{c}}_{nk}^{†}$ and ${\hat{c}}_{nk}$ (${\hat{a}}_{q\nu }^{†}$ and ${\hat{a}}_{q\nu }$) are the operators of the creation and annihilation of electrons (phonons), respectively. The core of ${\hat{H}}_{ep}$ is the EPI matrix element *g_mnv_* (*k*,*q*)—this matrix element quantifies the interaction strength between an electron with wavevector *k* and band *n*, and a phonon with wavevector *q* and branch *v*. The physical origin is the disturbance of electronic potential energy caused by lattice vibration [73, 74, 75].

EPI introduces additional thermal resistance to affect κ_ph_ by enhancing phonon scattering and shortening phonon lifetime. EPI can induce phonon self‐energy, expressed as:

(11) $$\begin{array}{c} \Pi_{\nu }\left(q,\omega_{q\nu }\right)=2\sum_{m,n}\int \frac{dk}{\Omega_{\mathrm{BZ}}}|g_{mn\nu }\left(k,q\right){|}^{2}\frac{f_{mk+q}-f_{nk}}{\varepsilon_{mk+q}-\varepsilon_{nk}-\hbar \omega_{q\nu }-i\delta } \end{array}$$

where *f*_*n*k_ is the distribution function for electrons. Ω_BZ_ is the volume of the first Brillouin zone, and δ is an infinitesimal regularization factor. It generates the imaginary part of the phonon self‐energy ImΠν(q,ωqν), which is directly proportional to the electron‐phonon scattering rate 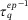 which can be given by:

(12) 1τqep=2ImΠνq,ωqνℏ=4πℏ∑mnνgmnνk,q2fnk−fmk+q×δεmk+q−εnk−ℏωqν

This scattering effect is particularly significant in high‐carrier‐concentration systems [76, 77, 78, 79, 80]. In addition, EPI can also change the phonon group velocity through phonon frequency renormalization. And the Kohn anomaly [81] (abrupt change of phonon dispersion at a specific wave vector) caused by EPI will further enhance the scattering of specific phonon modes, leading to a significant suppression of phonon thermal conductivity. In terms of electronic thermal conductivity regulation, EPI changes the magnitude and temperature dependence of κ_e_ by affecting the electron scattering mechanism and transport integral. It reduces the electrical conductivity by shortening the electron relaxation time, thereby indirectly reducing κ_e_ [82, 83].

Therefore, electron‐phonon coupling regulates the thermal conductivity of materials through three mechanisms: “enhanced phonon scattering”, “phonon frequency renormalization”, and “electronic transport modulation”. Its action strength, and mechanism can be accurately regulated through material composition design (such as doping, alloying), structural regulation (such as low‐dimensionalization, Fermi surface engineering), and external field regulation (such as temperature, magnetic field), providing a core theoretical basis for the thermal transport of UHTCs.

### 2.4 Thermal Radiation Theory

Thermal radiation is also a non‐negligible way of heat transfer at rising temperatures. The source of the emitted radiation is a combination of electronic and molecular oscillations and transitions in the emitting material, as well as lattice vibrations, as shown in Figure 3f. Once the energy is radiated, it propagates as an electromagnetic (EM) wave, regardless of whether there is a vacuum or matter along its path [84]. All objects above 0 K will emit thermal radiation, and both the spectral distribution and intensity are strongly governed by the temperature of the emitting object [84]. Blackbody is an idealized system that perfectly absorbs all incident electromagnetic energy and emits the maximum possible energy among all objects at a given temperature. The emission spectral distribution of a blackbody is only determined by its temperature according to Planck's law:

(13) $$E_{\lambda ,b}=\pi I_{\lambda ,b}\left(\lambda ,T\right)=\frac{2\pi hc_{0}^{2}}{\lambda^{5}\left[\left(exp(\frac{hc_{0}}{\lambda k_{B}T}\right)-1\right]}\;$$

where E_λ,b_ is the spectral emissive power, h is Planck's constant, λ is a certain wavelength, and c_0_ is the light speed in vacuum.

Through integrating the spectral emissive power E_λ,b_ over the whole electromagnetic spectrum, Stefan–Boltzmann's law gives the total radiative power E_b_ of a blackbody determined by temperature:

(14) $$E_{b}=\sigma T^{4}$$

here, σ is the Stefan–Boltzmann constant, and the equation indicates that the total energy of radiation increases with the fourth power of temperature.

While blackbody radiation provides an idealized benchmark, real materials act as less efficient thermal emitters. Their radiative performance is characterized by emissivity ε_em_, defined as the ratio of the emitted power of a material to that of a blackbody at the same temperature. The total emissive power of a real object is:

(15) $$E_{nb}=\varepsilon_{em}\;\sigma T^{4}$$

The emissivity of materials can be understood from a quantum mechanical perspective [85]. As for light incident on a material surface, three primary processes occur: reflection R, absorption α and transmission τ (α+R+τ = 1) in Figure 3g. The complex refractive index N and the complex permittivity ε are typically used to describe the macroscopic optical properties of objects [86].

(16) N=n+jk

(17) $$\varepsilon =\varepsilon^{\prime }+j\varepsilon^{\prime \prime }=\left(n^{2}-k^{2}\right)+j\left(2\mathrm{nk}\right)$$

where n represents the real part of the complex refractive index, and k denotes the extinction coefficient, characterizing energy absorption. And for the complex permittivity, the real part ε′ governs phase velocity and polarization, while the imaginary part ε′′ characterizes energy absorption. ε′′ arises from the absorption of photons that excite electrons from the valence band (VB) to the conduction band (CB). This process is formalized by Fermi's Golden Rule, which states that the absorption rate is proportional to the joint density of states and the transition probability matrix element. Subsequently, ε′ is determined by the complete knowledge of $\varepsilon^{\prime \prime }$ across all frequencies. So based on the Kramers–Kronig dispersion relation, the real and imaginary parts of the complex permittivity can be respectively calculated according to []:

(18) $$\begin{array}{ccc} \varepsilon^{\prime } & = & 1+\frac{8\pi^{2}e^{2}}{m^{2}}\sum_{V,C}\int_{BZ}d^{3}k\frac{2}{2\pi }\times \frac{{\left|\vec{e}\cdot \vec{M_{CV}}\left(\vec{K}\right)\right|}^{2}}{\left[E_{C}\left(\vec{K}\right)-E_{V}\left(\vec{K}\right)\right]}\\ & & \times \frac{h^{3}}{{\left(2\pi \right)}^{3}}\left[E_{C}\left(\vec{K}\right)-E_{V}\left(\vec{K}\right)-h^{2}\omega^{2}/{\left(2\pi \right)}^{2}\right] \end{array}$$

(19) $$\begin{array}{ccc} \varepsilon^{\prime \prime } & = & \frac{4\pi^{2}}{m^{2}\omega^{2}}\sum_{V,C}\int_{BZ}d^{3}k\frac{2}{2\pi }{\left|\vec{e}\cdot \vec{M_{CV}}\left(\vec{K}\right)\right|}^{2}\\ & & \times \delta \;\left[E_{C}\left(\vec{K}\right)-E_{V}\left(\vec{K}\right)-h\omega /\left(2\pi \right)\right] \end{array}$$

where BZ is the first Brillouin zone, $\vec{K}$ is a reciprocal lattice vector, ω is the angular frequency, ${|\vec{e}\cdot \vec{M_{CV}}\left(\vec{K}\right)|}^{2}$ is the momentum transition matrix element, and $E_{C}\left(\vec{K}\right)$ and $E_{V}\left(\vec{K}\right)$ are the intrinsic energy level of the conduction and valence bands, respectively. Then the full ε value can be used to predict the reflection R according to the Fresnel equation:

(20) $$R\;=\left[{\left(n-1\right)}^{2}+k^{2}\right]\;/\left[{\left(n+1\right)}^{2}+k^{2}\right]$$

Under thermal equilibrium, Kirchhoff's law ensures that emissivity equals absorptivity, according to which the opaque solids, emissivity can be calculated by:

(21) $$\varepsilon_{em}=\alpha =1-R$$

Thus, from the basic electronic structure of a material, the electronic band energy level and corresponding wavefunctions will indicate the full complex permittivity ε and then provide a complete physical description of the material's optical properties.

However, some UHTCs, like ZrB_2_, are metallic conductive materials with free electrons whose infrared radiation characteristics can be quite different. As for the dielectric function of such materials, considering only the interband transitions is clearly insufficient, especially in the low‐energy infrared region. Therefore, the dielectric function should comprehensively incorporate both intraband and interband transition effects for free electrons according to the Drude model [88]. In the low‐energy infrared region, since the frequency of incident light is lower than the plasma frequency, the free electrons inside the material undergo forced oscillation under the influence of the external field, generating longitudinal waves. As transverse electromagnetic waves, the incident light cannot interact with these longitudinal waves. Consequently, the materials exhibit a metallic high‐reflection characteristic toward low‐energy incident light, also explaining the weak intrinsic emissivity of many UHTC materials within the infrared wavelength range. Besides, with a quite shallow skin depth on the order of nanometers to micrometers, the thermal radiation in UHTCs is a surface‐confined phenomenon, meaning the radiation originates predominantly from the near‐surface region instead of the interior bulk.

Radiative properties are also influenced by factors such as surface roughness, impurity levels, film thickness, and so on. When it comes to the ultra‐high temperature environment, the surface radiative properties may be influenced by the high‐temperature chemical transformations and texture change. For example, Li et al. [89] studied ZrB_2_–SiC ceramics, showing that oxidization at elevated temperatures leads to silica formation and surface roughening, both of which increase emissivity compared to vacuum conditions. Such temperature‐ and environment‐dependent effects highlight both the challenges and opportunities in tailoring the surface radiative properties of UHTCs. Optimizing these characteristics is crucial for enhancing their stability and thermal performance under extreme conditions.

In the preceding sections, we primarily described heat transport in dense systems, focusing on the intrinsic behavior of the material. For practical systems containing porous structures, we briefly discuss here the case of porous structures, where the presence of pores introduces additional considerations. Compared to their dense counterparts, the heat conduction mechanisms in porous composites are more complex. It is no longer governed solely by lattice vibration conduction, but involves coupled effects including solid‐phase conduction, gas‐phase transport, and high‐temperature radiation.

Effective Medium Theory [90, 91] serves as a core bridge linking the microscopic pore structure to macroscopic effective properties. Its fundamental concept is to approximate a complex multiphase porous material as a homogeneous effective medium, characterized by a set of “effective” parameters that describe its average behavior. At the microscopic scale, when the characteristic size of the porous structure approaches the mean free path of gas molecules, the Knudsen effect becomes significant, thereby suppressing gaseous thermal conduction [92, 93, 94, 95, 96].

Besides, the direct surface‐to‐surface radiative exchange, governed by the wall temperatures and emissivity, introduces an additional heat flux which is macroscopically indistinguishable from conductive heat flow. At rising temperatures, researchers have shown a strong dependence of the effective thermal conductivities (ETCs) for the porosity samples, indicating that the contribution of radiative heat transfer was dominating [97]. This radiative contribution is often represented as an equivalent radiative conductivity that adds to the solid conductivity [98].

The heat transport theory elaborated above provides a solid foundation for understanding the microscopic mechanisms of phonon and photon transport in ultra‐high temperature ceramics. In the following sections, we will employ the theoretical framework developed in this chapter as an analytical tool, focusing on typical ultra‐high temperature ceramic material systems to systematically describe their thermal conductivity and radiative properties. Through this analysis of the thermophysical properties of specific materials, we aim to establish a comprehensive understanding that bridges microscopic mechanisms with macroscopic performance.

## 3 Heat Conduction in UHTCs

Under extreme conditions of ultra‐high temperature and high transient heat flux coupling, the thermal properties of materials directly determine the thermal management performance, thermal shock resistance, and structural failure threshold. For example, the surface temperature of the nose cone of a hypersonic aircraft can exceed 2300 K during reentry, with a heat flux exceeding 100 W/cm^2^ [99], requiring materials with thermal conductivity higher than 60 Wm^−1^K^−1^ to quickly dissipate the heat generated by aerodynamic heating and avoid catastrophic peeling caused by local melting or thermal stress accumulation. Therefore, the thermal conductivity of UHTCs is the core performance indicator for their application in extreme thermal environments, such as hypersonic aircraft thermal protection systems, shielding materials for nuclear fusion reactors, and rocket engine nozzles [4, 100, 101]. However, the thermal conductivity of UHTCs is inherently complex. It depends not only on intrinsic phonon transport and electron‐phonon interactions but also on defect/grain boundary densities, and dynamic environmental factors (e.g., oxidation and radiation damage at elevated temperatures), making it a focus of interdisciplinary research on UHTCs. These microstructural features enhance phonon scattering, markedly suppressing thermal conductivity.

Furthermore, the contribution of electronic thermal conductivity in UHTCs is significant (often exceeding 30%), necessitating its analysis for accurate thermal transport interpretation. Experimentally, the Wiedemann‐Franz law is typically used to decouple electronic (κ_e_) and phononic (κ_ph_) contributions. Yet this approach suffers significant limitations—the Lorenz number exhibits strong temperature‐ and material‐dependence, frequently leading to underestimation of κ_ph_ [36, 37, 102] and even nonphysical negative κ_ph_ values [103, 104].

Recent advances integrating computational materials science with advanced characterization are accelerating understanding of UHTCs’ thermal transport. First‐principles calculations analyze phonon spectra and electron density of states contributions [49], while solutions to the BTE parameterized by Density Functional Theory (DFT) predict intrinsic thermal conductivity from spectral phonon/electron data [105, 106, 107, 108]. (Equation 5, Section 2). Molecular Dynamics (MD) simulations—using Green‐Kubo or Non‐Equilibrium MD (NEMD) methods—extract thermal conductivity by tracking atomic trajectories, offering advantages for polycrystalline/defect systems but limited by interatomic potential accuracy [109, 110, 111]. Experimentally, transient laser thermal conductivity meters and laser flash rules provide high‐precision experimental methods for extreme thermal performance testing [112]. This section systematically reviews recent progress in theoretical and experimental understanding of thermal transport in various UHTCs including borides, carbides, and nitrides, covering prediction and measurement results, and theory‐experiment comparisons.

### 3.1 Borides

Borides, due to their unique properties such as high melting points, excellent high‐temperature stability, oxidation resistance, good thermal conductivity, and chemical inertness, have been extensively studied for their thermal properties, with HfB_2_ and ZrB_2_ being two highly representative ones.

**FIGURE 4:**
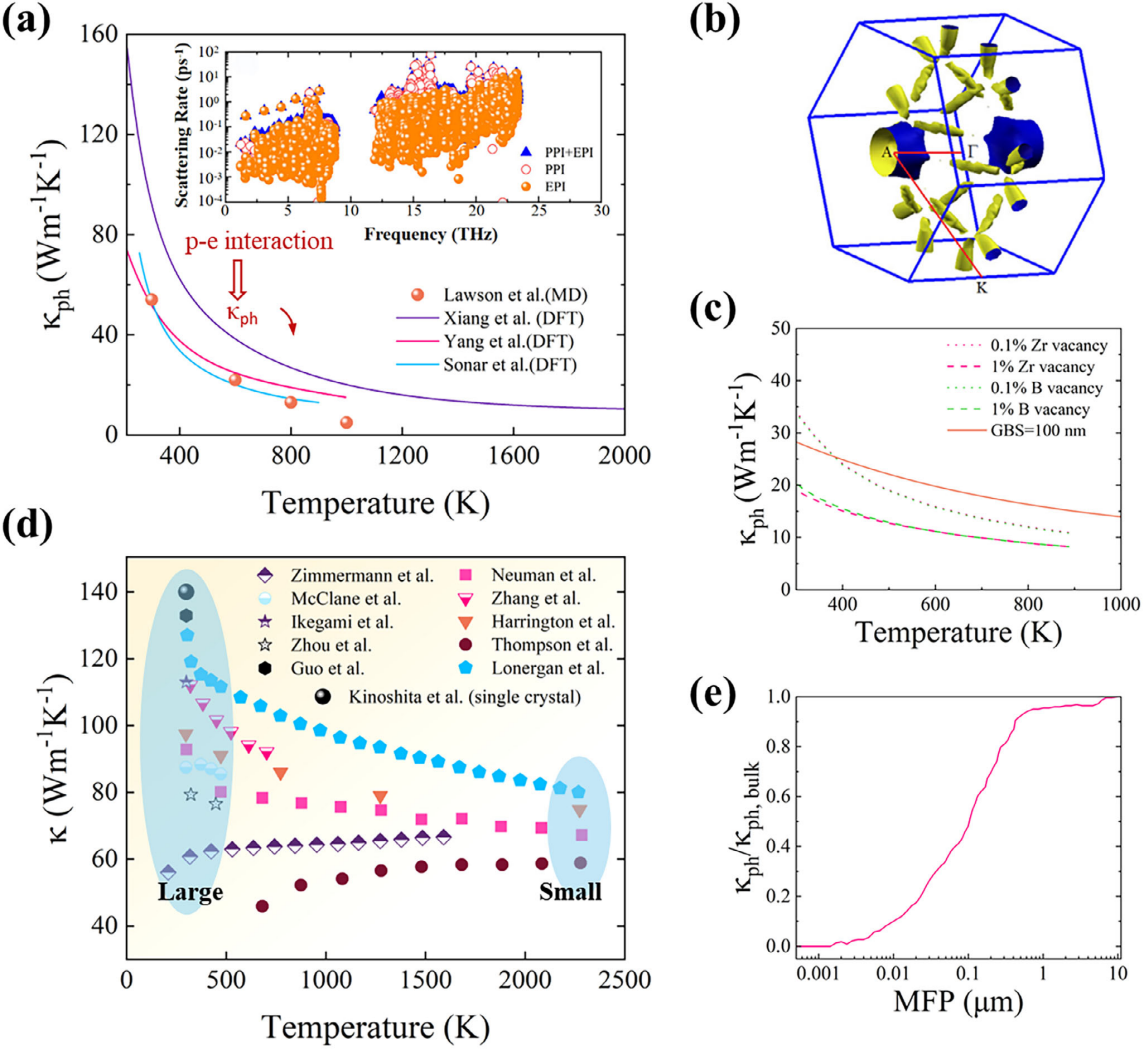
Thermal transport properties in ZrB_2_. (a) Theoretical calculation of κ_ph_ results for ZrB_2_ [113, 114, 115, 116]. Inset is the scattering rates for different scattering modes [115]. (b) The Fermi surfaces of ZrB_2_. Reproduced with permission [115]. Copyright 2020, Elsevier. (c) The influence of vacancies and grain size on the thermal conductivity of ZrB_2_ [115, 116]. (d) Multiple sets of experimental [29, 35, 36, 37, 102, 103, 104, 112, 117, 118, 119] thermal conductivity results for ZrB_2_. (e) Contribution of phonon mean free path to thermal conductivity at 300 K [116].

Figure 4a shows the calculated phonon thermal conductivity of ZrB_2_ vs. temperature by several research teams [113, 114, 115, 116]. Among them, except for Lawson et al. [113], who used MD simulations, all other studies were conducted through first principles. Most of the calculation results [113, 115, 116] obtained at room temperature have a κ_ph_ of about 50 Wm^−1^K^−1^, indicating a high overall consistency. It is worth noting that the calculation results of Xiang et al. [114] are significantly higher than those of other research groups, and their values are similar to the predicted values in Yang et al. [115]’s study, which did not consider electron‐phonon interactions. This indicates that Xiang et al.’s calculations may not have fully taken into account the influence of phonon scattering by electrons. As shown in Figure 4b, the “folded dumbbell‐shaped” hole surface of ZrB_2_ is nested with the circular electron surface, resulting in strong electron‐phonon interactions. As shown in Figure 4a, the phonon scattering contribution dominated by electron‐phonon interactions accounts for 38%–52% at 300 K, resulting in a decrease in κ_ph_ from 79 to 49 Wm^−1^K^−1^ [115]. However, all studies have not taken into account the influence of four‐phonon scattering, which may lead to an overestimation of κ_ph_.

So far, the calculation of the electronic thermal conductivity of ZrB_2_ has not been fully studied. However, it can be inferred that the value of κ_e_ is non‐negligible in ZrB_2_, since the thermal conductivity from most experiments was substantially higher than the calculated κ_ph_ as shown in Figure 4d. It can also be observed that the reported thermal conductivity values fluctuate greatly, with a range of 53–145 Wm^−1^K^−1^ at room temperature. However, at the high temperature (2300 K), the range of thermal conductivity is 50–80 Wm^−1^K^−1^. The variance in thermal conductivity gradually diminishes as the temperature increases. This trend is consistent with that shown in Figure 3e, as described in Chapter 2. The underlying reason is that the rise in temperature leads to an increase in the number of phonons. Although this also enhances phonon scattering by defects and boundaries, the increase in phonon–phonon scattering is even more pronounced. Consequently, at elevated temperatures, thermal transport is dominated by phonon–phonon scattering. Thus, the differences in thermal conductivity observed at room temperature—primarily stemming from phonon scattering by defects and boundaries—gradually diminish as the temperature rises. Except for Zimmermann et al. [36], the room temperature thermal conductivity is above 80 Wm^−1^K^−1^. Kinoshita et al. [35] prepared ZrB_2_ single crystals using the radio frequency heating floating zone method and tested their thermal properties when studying ZrB_2_ as a base for GaN lattice matching. The thermal conductivity exhibits anisotropy, with a thermal conductivity of 132–145 Wm^−1^K^−1^ parallel to the a‐axis and 95–102 Wm^−1^K^−1^ parallel to the c‐axis. Unfortunately, ZrB_2_ single crystals were only evaluated for their low‐temperature thermal conductivity. The thermal conductivity of single‐crystalline ZrB_2_ at high temperatures has not been experimentally reported yet. The results of Zimmermann et al. [36] and Thompson et al. [102] show significant differences from other studies, with thermal conductivity increasing with temperature. The reason for this phenomenon is the use of WC during the grinding process. The introduction of W forms a (Zr, W)B_2_ solid solution, which introduces additional scattering and consequently reduces the thermal conductivity [103, 120]. Meanwhile, W doping also affects the electronic transport behavior of ZrB_2_, leading to an increase in electronic thermal conductivity with rising temperature [103, 121]. The combined effect of these two mechanisms gives rise to the observed thermal conductivity behavior. Lonergan et al. [29] obtained a high thermal conductivity value, which may be related to the degree of densification and grain size of the sample: the relative densities and grain size of the sample is 99.5% and 25 µm. From room temperature to 2273 K, the thermal conductivity decreases from 127 to 80 Wm^−1^K^−1^. Guo et al. [119] achieved a very high value, with a thermal conductivity of 133 Wm^−1^K^−1^ at room temperature. However, the measured specific heat capacity was overestimated by about 25%, and the relative density was low. The actual thermal conductivity is approximately 100 Wm^−1^K^−1^. The polycrystalline ZrB_2_ reported by Lonergan et al. [29] is closest to the thermal conductivity of a single crystal, and represents the intrinsic thermal conductivity of polycrystalline ZrB_2_.

To identify the key influencing factors, investigating the roles of grain size and internal defects is also of great importance. As described in Chapter 2, the presence of vacancies, defects, and grain boundaries increases phonon scattering, thereby affecting heat transfer. Figure 4c,e illustrate the effects of vacancy concentration [116], grain size [115], and phonon mean free path [116] on the phonon thermal conductivity. At 300 K, a 0.1% vacancy concentration in Zr and B reduces κ_p__h_ to 34.6 and 33.8 Wm^−1^K^−1^, respectively. When the vacancy concentration increases to 1%, κ_p__h_ further decreases to 19 and 20.3 Wm^−1^K^−1^. Meanwhile, when the grain boundary spacing is 100 nm, κ_p__h_ decreases to 29 Wm^−1^K^−1^. The influence of grain size on phonon mean free path can be more clearly observed from the cumulative mode curve of κ_ph_ as shown in Figure 4e: only when the mean free path approaches 1 µm, the thermal conductivity has reached 95% of the bulk. However, the suppression of thermal conductivity can also be observed in experiments where the grain size of zirconium diboride are greater than 1 µm [29, 36, 102, 103, 104, 112, 117, 118, 122, 123], due to the existence of interfacial thermal resistance. For example, when the grain size is on the order of several micrometers, the interfacial thermal resistance of typical ceramics is approximately 10^−7^ m^2^K/W [124, 125, 126, 127, 128, 129]. Based on a thermal conductivity of 100 Wm^−1^K^−1^, the corresponding equivalent thermal resistance is estimated to be on the order of 10^−7^ m^2^K/W when the grain size is ∼10 µm. Thus, the interfacial thermal resistance is comparable to the intrinsic thermal resistance of the grain and is a factor influencing thermal conduction. Furthermore, almost all samples are not fully dense. Even when a sample can be synthesized to near full density, internal imperfections such as second phases and defects inevitably remain. As indicated by the calculations above, these scattering mechanisms reduce thermal conductivity. Consequently, the combined presence of grain boundaries, defects, vacancies, and second phases in polycrystalline samples leads to significant fluctuations in the thermal conductivity of ZrB_2_.

**FIGURE 5:**
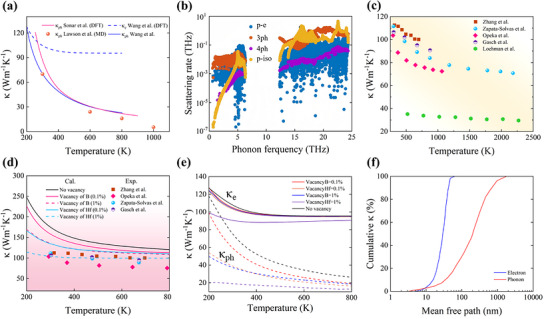
Thermal transport properties in HfB_2_. (a) Theoretical calculation of k_ph_ and k_e_ results for HfB_2_ a‐axis [49, 113, 116]. (b) Scattering rate results for different scattering modes in HfB_2_. Reproduced with permission [49]. Copyright 2023, Youke Publishing. (c) Multiple sets of experimental [30, 37, 130, 131, 132] thermal conductivity results for HfB_2_. (d) Comparison of thermal conductivity in HfB_2_ between calculation [49] (lines) and experiments (symbols). (e)Effects of HfB_2_ vacancies on the thermal conductivity of phonon (red) and electron (blue) [49]. (f) Cumulative thermal conductivity relative to the mean free path of HfB_2_ [49].

Figure 5a shows the comparative results of HfB_2_ κ_ph_ and κ_e_ in different studies [49, 113, 116]. At room temperature, the κ_ph_ is about 70 Wm^−1^K^−1^. Wang et al. [49] conducted research on the electronic thermal conductivity of HfB_2_ and found that the κ_e_ values of the a‐ and c‐axis were 104.5 and 88.5 Wm^−1^K^−1^, respectively, at 300K. This anisotropy is due to the higher electron group velocity in the a‐axis direction of the HfB_2_ with a hexagonal crystal structure, where the electron band dispersion relationship is steeper, resulting in a significantly higher average group velocity than the c‐axis. The κ_e_ shows a trend of decreasing first and then tending to be constant with increasing temperature. At low temperatures (< 300 K), as the temperature increases, the number of phonons occupied increases following the Bose‐Einstein distribution, leading to an increase in electron‐phonon scattering rates. However, the specific heat of electrons has not significantly increased at this time, leading to a decrease in κ_e_ with increasing temperature. In the high‐temperature region (> 500 K), the electron specific heat increases with temperature, competing with the scattering rate, which results in the κ_e_ being nearly temperature‐independent. It is worth noting that in the study by Wang et al. [49], the total thermal conductivity of the a‐ and c‐axis at 300K was 175 and 157.7 Wm^−1^K^−1^, respectively. The contribution of electrons reached 59.4% and 54.2%, and this proportion further increased with temperature. At 800 K, the total thermal conductivity was 121 Wm^−1^K^−1^ along the a‐axis and 108 Wm^−1^K^−1^ along the c‐axis. The electronic contribution dominated the heat conduction in both directions, comprising 79% of the total for the a‐axis and 80% for the c‐axis. On the other hand, the electron‐phonon effect is equally important in HfB_2_, as shown in Figure 5b. At room temperature, the electron‐phonon scattering of low‐frequency phonons has a high scattering rate, and low‐frequency phonons mainly transfer heat. It can be noted that phonon‐isotope scattering also has a high scattering rate, which cannot be ignored in calculations. Compared to the above scattering, the four‐phonon scattering effect is not significant, but at high temperatures, its effect increases and becomes an undeniable influence in high‐temperature thermal transport.

Figure 5c shows the thermal conductivity of experimentally synthesized HfB_2_ as a function of temperature. The κ of HfB_2_ presents a similar temperature‐dependent trend overall, with differences between different research groups. In existing reports, the room temperature thermal conductivity is generally higher than 100 Wm^−1^K^−1^, but Loehman et al. [130] reported a lower value (35 Wm^−1^K^−1^ at 523 K), which is closely related to the small specific heat capacity parameter used and the low degree of sample densification (68%). The highest reported HfB_2_ thermal conductivity [37] (112 Wm^−1^K^−1^) comes from nearly fully dense samples prepared from high‐purity raw materials (relative density 98.1%). In addition, the existing HfB_2_ thermal conductivity data are based on polycrystalline samples, and the intrinsic thermal transport characteristics of single‐crystal materials still lack systematic evaluation. It is worth noting that even with the highest experimental value [37] (112 Wm^−1^K^−1^), there is still a significant difference compared to the theoretically predicted value [49] (> 160 Wm^−1^K^−1^), indicating the inhibitory effect of grain boundary scattering and defect states on thermal conductivity.

Next, we compare the experimental values with the first principle calculations [49], as shown in Figure 5d. The experimental values are significantly lower than those calculated by DFT, and the difference is mainly due to microscopic defects in polycrystalline materials. The core root of this difference lies in the microstructural defects (such as vacancies and interstitial atoms) commonly present in actual materials and the strong scattering effect of grain boundary networks on phonon and electron transport. This difference can be reduced by over‐introducing vacancies: the inhibitory effect of Hf vacancies on κ_e_ and κ_ph_ is stronger than that of B vacancies. As shown in Figure 5e, 1% Hf vacancies can reduce κ_e_ to 99 and 90 Wm^−1^K^−1^ at 200 and 800 K, respectively, and decrease κ_ph_ to 20 and 12 Wm^−1^K^−1^, respectively [49]. When the Hf vacancy concentration reaches 1%, the calculated thermal conductivity is consistent with the experimental value (Figure 5d). As the main source of phonon scattering, the strength of grain boundaries depends on the relative relationship between the phonon MFP and grain size. As shown in Figure 5f, the phonon MFP distribution range of HfB_2_ at 300 K is mostly 100–1000 nm, significantly larger than that of the electronic MFP. The MFP that contributes 50% of phonon thermal conductivity is still much higher than that of electrons, indicating that miniaturization has a more significant inhibitory effect on phonon thermal transport.

In the above discussion, we have highlighted the significant role of electron‐phonon coupling in influencing the thermal transport behavior of borides. However, the κ_dif_ described in Chapter 2 has not been fully addressed in this section. The primary reason is that systematically distinguishing the contribution of κ_dif_ from other heat transfer mechanisms to the total thermal conductivity requires thermal property data from single‐crystal samples over a wide temperature range. Currently, due to the scarcity of UHTC single crystals, it remains challenging to accurately assess whether κ_dif_ dominates the high‐temperature thermal transfer process in these materials or how it couples with other mechanisms. In other words, although existing polycrystalline samples are well‐prepared and yield reliable data, the presence of grain boundaries, defects, and other microstructural features introduces additional scattering mechanisms that may obscure or interfere with intrinsic phonon diffusive transport behavior. Therefore, the lack of single‐crystal thermal property data limits our ability to gain a fundamental physical understanding of the actual role of κ_dif_ in UHTCs. So, one key direction for future research should focus on the synthesis of single crystals and the characterization of their thermophysical properties over a wide temperature range, particularly under extremely high‐temperature conditions.

### 3.2 Carbides

**FIGURE 6:**
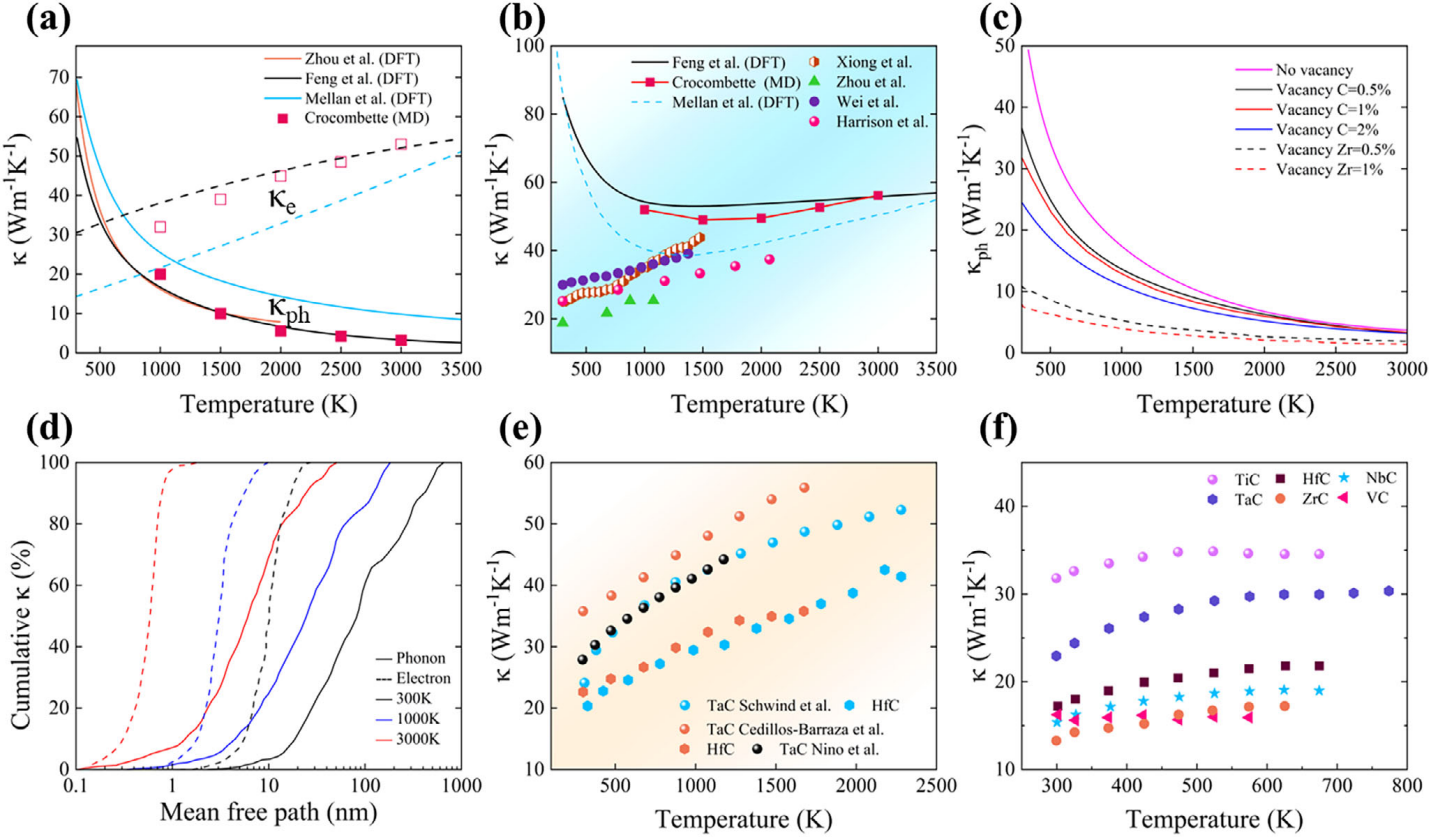
Thermal transport properties of carbides. (a) The calculation results of ZrC thermal conductivity show that the solid line represents the phonon contribution and the dashed line represents the electron contribution [70, 133, 134, 135]. (b) Calculation [70] and experimental [24, 138, 139, 140] comparison of the thermal conductivity of ZrC. (c) The influence of vacancies on the thermal conductivity of ZrC [70]. (d) Cumulative thermal conductivity of ZrC under different mean free paths [70]. (e) Thermal conductivity of TaC and HfC [25, 26, 141]. (f) Thermal conductivity of transition metal carbides [26].

Carbides are not only one of the components with the highest melting point in UHTCs, but also possess unique electronic and phonon transport behaviors due to their own characteristics. Figure 6a shows the evolution of ZrC thermal conductivity with temperature in different calculations [70, 133, 134, 135]. Unlike boride systems, existing research on carbides have covered the ultra‐high temperature range (> 3000 K), all calculations show consistent room temperature κ_ph_ of ∼60 Wm^−1^K^−1^, and typical negative temperature dependence of crystals. Feng et al.’s [70] calculations indicate that the contribution of phonons drops sharply from 55 Wm^−1^K^−1^ at 300 K to 2.9 Wm^−1^K^−1^ at 3500 K, consistent with the MD simulation results obtained by Crocombette [133]. The strong temperature dependence is due to the dual scattering effect: (1) Three‐phonon scattering dominates the low‐temperature range: lattice vibration anharmonicity led to phonon‐phonon scattering increasing with temperature, shortening the phonon mean free path; (2) Activation of four‐phonon process at high‐temperature: after exceeding 2000 K, higher‐order four‐phonon scattering processes significantly increase, further suppressing phonon transport efficiency. It is worth noting that various studies have observed a unique behavior of ZrC electronic thermal conductivity κ_e_ that increases monotonically with temperature. All calculations consistently indicate that κ_e_ increased from 30 Wm^−1^K^−1^ at 300 K to 54 Wm^−1^K^−1^ at 3500 K. This abnormal heating behavior can be attributed to the two mechanisms related to its semimetallic electronic structure effect [136, 137]: The spin polarization band of ZrC exhibits a metal‐like density of states distribution at the Fermi level, and its Lorenz number increases with temperature, directly leading to an enhancement of electronic thermal conductivity. Second, as the temperature increases, the density of states (DOS) of the Fermi level also increases. In practical materials, vacancies also have an impact on this phenomenon. At high temperatures, the concentration of lattice vacancies increases, introducing temperature‐independent carrier scattering channels. Combined with Wiedemann–Franz law, a temperature dependent electron transport enhancement effect is formed. In addition, with increasing vacancy concentration, the DOS near the Fermi level shows an upward trend, and the electron scattering rate synchronously increases, resulting in two opposite effects that may also lead to an increase in κ_e_.

Figure 6b shows the comparison of the thermal conductivity of ZrC between experiments [24, 138, 139, 140] and calculations. At room temperature, the experimentally measured thermal conductivity falls within the range of 19–30 Wm^−1^K^−1^, which deviates considerably from the theoretical value of 85 Wm^−1^K^−1^. However, this discrepancy gradually diminishes as the temperature increases. For instance, in the study by Xiong et al. [24], the thermal conductivity reaches 44 Wm^−1^K^−1^ at 1473 K, compared with the calculated value of 53 Wm^−1^K^−1^. Zhou et al. [140] low thermal conductivity (20 Wm^−1^K^−1^ at room temperature) is directly correlated with insufficient densification degree (91.9%), and pore‐induced phonon‐defect scattering that reduces phonon thermal conductivity.

To explore the specific reasons for the low thermal conductivity observed in the experiment, Feng et al. [70] investigated the variation of ZrC thermal conductivity with mean free path and the influence of vacancies on thermal conductivity. Figure 6c shows that C and Zr vacancies reduce the phonon thermal conductivity to different extents. At 300 K, 1% and 2% of C vacancies reduce κ_ph_ to 32 and 24 Wm^−1^K^−1^, respectively, while only 0.5% of Zr vacancies can cause κ_ph_ to decrease to 10 Wm^−1^K^−1^ [70]. This phenomenon better explains why the thermal conductivity of carbides increases with temperature in the experiment. κ_ph_ is greatly affected by defects, while κ_e_ is less affected. Thus, electrons dominate thermal conductivity, and κ_e_ shows an upward trend with temperature, leading to an overall increase in thermal conductivity with temperature. At high temperatures, the contribution of phonons decreases, resulting in smaller differences from theoretical calculations at high temperatures. Figure 6d shows that at 300, 1000, and 3000 K, 80% of κ_ph_ in ZrC is contributed by phonons with MFP < 300, 60, and 20 nm, respectively, which means the grain boundary scattering effect is more prominent at low temperatures (< 300 K). In contrast, 80% of the contribution of κ_e_ comes from electrons with MFP < 15 nm at 300 K, and the MFP becomes even less at elevated temperatures, indicating that the scattering intensity of phonons by grain boundaries is much higher than that of electrons. In summary, the thermal properties of UHTCs are significantly influenced by grain size, an effect that diminishes with increasing temperature. It is important to note that this influence is not the reason experimental values are lower than computational results. This characteristic provides a new strategy for optimizing thermoelectric performance: via micro/nano‐fabrication, the material size can be engineered to a critical regime where the phonon MFP is larger than the grain size, which in turn is larger than the electron MFP. Through this approach, phonon transport can be effectively suppressed while preserving electronic conduction, thereby decoupling heat flow from electrical flow.

Similar to ZrC, other carbides also have similar thermal properties. As shown in Figure 6e, the thermal conductivity of HfC at room temperature is approximately 18–22 Wm^−1^K^−1^, reaching 42 Wm^−1^K^−1^ at 2300 K [25, 26, 141]. There is good consistency between Schwind et al. [141] and Nino et al. [142]’s reports on TaC, with Cedillo‐Barraza et al. [25] reporting slightly higher values. All three groups synthesized TaC with a high degree of densification (98%). The thermal conductivity of TaC at room temperature is around 30 Wm^−1^K^−1^, while Cedillo‐Barraza et al. [25] measured 56 Wm^−1^K^−1^ at 1700 K, slightly higher than Schwind et al.’s [141] 52 Wm^−1^K^−1^ at 2300 K. Liang et al. [26] measured the thermal conductivity of various transition metal carbides (Figure 6f) using the laser flash method. Like other experiments, all carbides exhibited anomalous behavior of increasing thermal conductivity with increasing temperature. This phenomenon, related to κ_e_ that increases with temperature, may exist throughout the entire transition metal carbide system.

Carbides not only exhibit unique electron thermal transport behavior, but some of them also exhibit electron‐phonon coupling effects. The phonon thermal conductivity of typical metallic and nonmetallic crystals decreases rapidly with increasing temperature, as the Umklapp scattering rates increase with temperature due to enhanced phonon populations. However, when phonon‐electron scattering dominates over the phonon‐phonon Umklapp scatterings, the κ_ph_ tends to saturate at high temperature. In their first‐principles calculations of transition metal carbides, Li et al. [50] found that in metals with nested Fermi surfaces and large frequency gaps between acoustic and optical phonons, the phonon thermal conductivity κ_ph_ is almost no longer affected by temperature. In this regime, the phonon‐electron interactions outweigh phonon‐phonon interactions, resulting in fundamentally different behavioral characteristics of κ_ph_.

Fermi surface nesting (FSN) can generate strong electron–phonon interactions by enabling large portions of the Fermi surface to be connected through specific wavevectors [72, 143, 144]. In a typical electron–phonon process, an electron scatters from one state to another while emitting or absorbing a phonon. As phonon energies are much smaller than the scale of electron energy, energy conservation requires that both electron states are close to the Fermi surface. Momentum conservation requires that the phonon wavevectors balance the two electron wavevectors. When the Fermi surface contains parallel or nearly parallel segments, many electron states can be connected by the same phonon wavevector, greatly enhancing electron‐phonon interactions.

**FIGURE 7:**
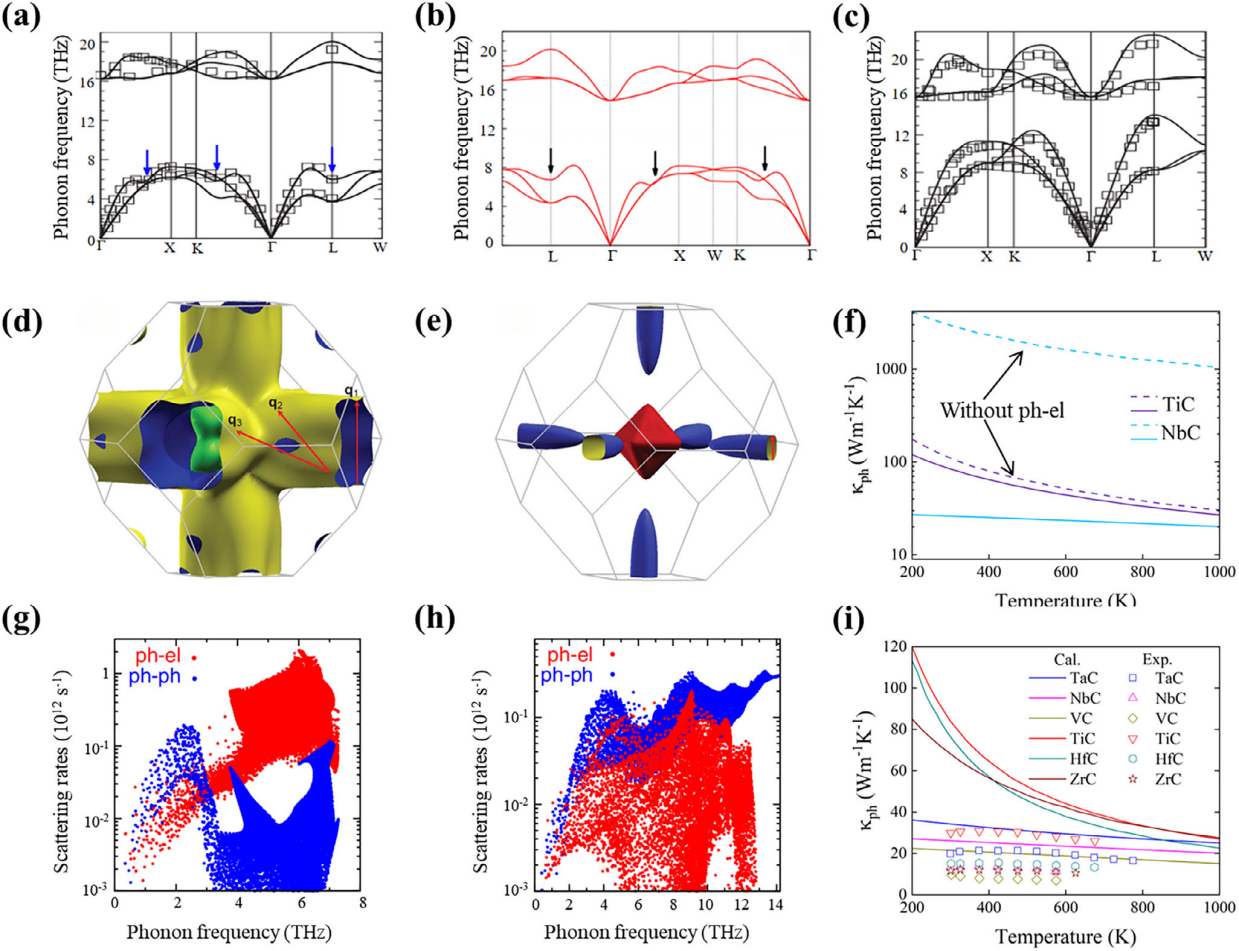
Strong phonon‐electron interactions in carbides [26, 50, 145, 146, 147, 148, 149, 150]. Phonon dispersion diagrams of NbC (a), VC (b), and TiC (c). The arrow indicates the anomalous phenomenon of phonons. And the black square represents experimental data. The Fermi surfaces of NbC (d) and TiC (e). The red arrows represent three nesting vectors. (f) The relationship between phonon thermal conductivity and temperature, with dashed lines representing phonon‐phonon scattering and phonon‐isotope scattering, and solid lines incorporating phonon‐electron scattering. Comparison of phonon‐phonon (blue) and phonon‐electron (red) scattering rates for (g) NbC and (h) TiC at 300 K. (i) Experimental and computational comparison of phonon thermal conductivity of transition metal carbides. Reproduced with permission [50]. Copyright 2018, APS.

When FSN coincides with specific phonon properties, for example, when a large energy gap between acoustic and optical phonons suppresses the scatterings between acoustic and optical phonons, it can lead to simultaneously weak phonon‐phonon interactions and strong phonon‐electron interactions, in contrast to conventional behavior. The strong electron‐phonon interaction makes the κ_ph_ almost unaffected by temperature at the high temperature limit. Figure 7a,b show the phonon dispersion spectra of NbC and VC, respectively. There is a very large gap between the acoustic and optical phonons, which is much larger than the band gap of TiC in Figure 7c. The black arrow indicates the anomalous phenomenon of phonons, while no anomalous phenomenon is observed in TiC. Figure 7d shows the calculated Fermi surface structure of NbC [50]. There are three nesting vectors along [100, 110, 111] high‐symmetry directions [red arrows in Figure 7d]. The nesting regions give rise to strong interactions between electrons and phonons, which renormalize some phonon energies, producing phonon anomalies [145, 146, 147, 148]. In contrast, TiC does not have such a large Fermi surface nesting (Figure 7e). Figure 7g,h visually demonstrate the comparison between the phonon‐phonon scattering rate and the phonon‐electron scattering rate of various UHTCs. In the low‐frequency range, the intensity of phonon‐phonon scattering is significantly higher than that of phonon‐electron scattering. As the frequency increases, the phonon‐phonon scattering rate significantly decreases in the 4–8 terahertz frequency range, which is due to the small phase space of phonon scattering in this frequency band. As FSN drives an increase in electron‐phonon scattering, phonon‐phonon scattering in TiC is always stronger than phonon‐electron scattering (Figure 7h), and without the drive of FSN, phonon‐electron scattering is very low. At the same time, Li et al. [50] found that transition metal carbides from group V (VC, TaC) have similar characteristics to NbC, while transition metal carbides from group IV (HfC, ZrC) have similar characteristics to TiC. The superposition of these two effects results in a weak temperature dependence of phonon thermal conductivity in V‐group transition metal carbide NbC (Figure 7f), while the transition metal carbides of group IV still exhibit the temperature dependence of traditional T^−1^ (Figure 7i). The phonon and electronic properties involved in this abnormally weak temperature dependence of κ_ph_ also exist in other compounds. ScC and YC [146] also exhibit FSN and have larger electron‐phonon coupling constants, and their phonon‐electron scattering effects should be very significant. However, the frequency difference between acoustic and optical phonons in these materials is not sufficient to completely suppress the scattering effect between phonons. It is expected that the temperature dependence of κ_ph_ in these compounds will be higher than that of the aforementioned materials, but still lower than that of traditional T^−1^.

To verify this anomalous characteristic, Liang et al. [26] conducted thermal conductivity tests on transition metal carbides, calculated the Lorentz number using the Sheard formula, and separated the κ_ph_ using the Wiedemann‐Franz law. As shown in Figure 7i, the κ_ph_ of all transition metal carbides exhibits weak temperature dependence, which deviates from the calculation results. The samples synthesized in the experiment are all polycrystalline materials, and their internal defects can affect the κ_ph_, and the κ_ph_ separated by the Wiedemann–Franz law also has certain errors. However, overall, Liang et al.’s experiments further confirm that this weak temperature dependence is due to strong electron‐phonon interactions and weak phonon interactions.

### 3.3 Nitrides

**FIGURE 8:**
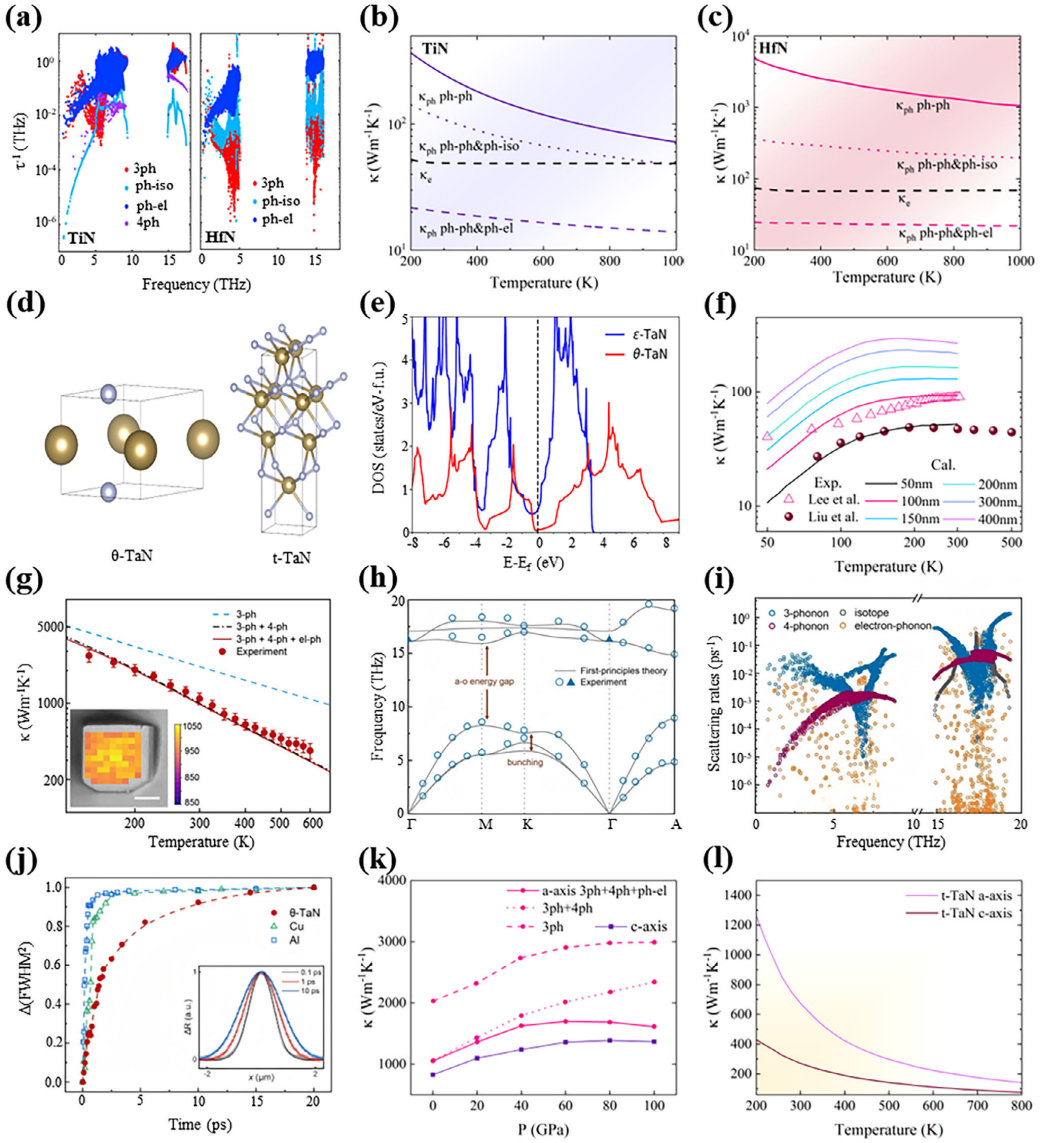
Thermal transport properties of nitrides. (a) Phonon scattering of TiN and HfN. Reproduced with permission [28]. Copyright 2020, Elsevier. Thermal conductivity of TiN (b) and HfN (c). (d) Crystal structure of θ‐TaN and t‐TaN. (e) Electron density of states for ε‐TaN (blue) and θ‐TaN (red). Reproduced with permission [154]. Copyright 2021, APS. (f) Thermal conductivity of θ‐TaN as a function of temperature from experiments [46, 156] (symbols) and calculations [46] with different grain sizes (lines) by taking into account all phonon scattering mechanisms. (g) Experimentally measured thermal conductivity of θ‐TaN in comparison to first‐principles calculations considering. Insets: 2D spatial mapping of thermal conductivity measured across the entire θ‐TaN crystals [51]. (h) Experimentally measured phonon band structure of θ‐TaN from IXS (circles) and Raman spectroscopy (triangles), overlaid with first‐principles calculations (solid lines) [51]. (i) Phonon scattering rates from first‐principles calculations [51]. (j) Time‐dependent electron relaxation profiles measured for θ‐TaN (red), Cu (green), and Al (blue), quantified by the squared full‐width at half‐maximum (FWHM^2^) of Gaussian fits (inset). Reproduced with permission [51]. Copyright 2026, AAAS. (k) Thermal conductivity of θ‐TaN varies with pressure [157]. (l) Thermal conductivity of t‐TaN along the a‐axis (purple) and c‐axis (brown) [158].

Strong electron‐phonon coupling is also present in nitride systems, and nitride materials with ultrahigh thermal conductivity [51] (> 1000 Wm^−1^K^−1^) have recently been discovered. Bao et al. [28] systematically revealed the thermal transport properties of typical metallic transition‐metal nitrides, TiN and HfN, through first principles calculations, exhibiting extremely high phonon thermal conductivity even above diamond (Figure 8b,c). For example, the κ_ph_ of HfN was estimated as high as 3335 Wm^−1^K^−1^ at 300 K. The κ_ph_ of TiN is lower than that of HfN, still reaching 243 Wm^−1^K^−1^. The ultrahigh thermal conductivity of HfN stems from the suppression effect on three‐phonon scattering induced by the unique phonon dispersion relations. (1) The atomic mass difference between the metal atom and the nitrogen atom forms a wide phonon energy gap, which remarkably hinders the “two acoustic phonons + one optical phonon” scattering process. (2) The phonon dip and softening phenomena of acoustic branches near the phonon peculiarities reduce the phase space for scattering between acoustic phonons [64]. (3) The narrowed optical phonon bandwidth further suppresses the “one acoustic phonon + two optical phonons” scattering [151]. However, phonon‐isotope scattering and phonon‐electron scattering have an effect on the phonon thermal transport of TiN and HfN. After introducing the natural isotope composition, the κ_ph_ of HfN drops sharply from 3335 to 319 Wm^−1^K^−1^, a decrease of one order of magnitude, while the κ_ph_ of TiN decreases to approximately 110 Wm^−1^K^−1^. Phonon‐electron scattering is the most critical mechanism determining the thermal transport of TiN and HfN. As shown in Figure 8a, the phonon‐electron scattering intensity in HfN is two orders of magnitude higher than that of conventional phonon‐phonon scattering. Similarly, in TiN, phonon‐electron scattering also exceeds phonon‐phonon scattering. After considering phonon‐electron scattering, the κ_ph_ of both materials drops drastically. This strong scattering originates from the ubiquitous FSN in TiN and HfN. Unlike the strong temperature dependence of three‐phonon scattering, phonon‐electron scattering exhibits weak temperature dependence, ultimately leading to the nearly temperature‐independent behavior of κ_ph_ in TiN and HfN, following the relations of κ_ph_∼T^−0.3^ and κ_ph_∼T^−0.09^, respectively. ZrN [152] also exhibits FSN and phonon anomalies, with a relatively large electron‐phonon coupling constant. In addition, the phonon dispersion curve of ZrN also shows a large band gap, indicating that this characteristic may also exist in ZrN. In general, the total thermal conductivity of TiN and HfN is co‐dominated by electrons and phonons. The electronic thermal conductivity of both materials is almost temperature‐invariant in the range of 200–1000 K, being 49 Wm^−1^K^−1^ for TiN and 69 Wm^−1^K^−1^ for HfN at 300 K. The proportion of phonon contribution is less, with the κ_ph_ of TiN accounting for 29% of the total thermal conductivity and that of HfN accounting for 26% at 300 K. In addition, the impact of the four‐phonon effect on the overall thermal conductivity is negligible at room temperature.

Inspired by the TiN and HfN, suppressing electron‐phonon scattering may be a means of developing UHTCs with ultra‐high thermal conductivity. Tantalum nitride (TaN) described below is a good example. TaN, as a typical representative of transition metal nitrides, has an ultra‐high melting point of ∼3090°C. Its crystal structure undergoes three‐phase evolution [153]: low‐pressure hexagonal ε‐TaN, high‐temperature low‐pressure cubic δ‐TaN, and recently synthesized θ‐TaN (Figure 8d). As a common phase in TaN, ε‐TaN has a large band gap in its phonon dispersion pattern and nested Fermi planes (Figure 8e) [154]. The large phonon energy gap usually leads to a high phonon thermal conductivity, but the presence of strong electron‐phonon interactions causes a sharp decrease in its phonon thermal conductivity (only 3–8 Wm^−1^K^−1^ [155]).

In recent years, theoretical research [154, 159] on θ‐TaN has revealed its groundbreaking thermal transport characteristics. First principles calculations show that this phase exhibits a unique frequency band gap in the phonon spectrum: there is a significant phonon energy gap, effectively suppressing the three‐phonon scattering process. Meanwhile, the low electron density of states near its Fermi level (Figure 8e) significantly reduces the strength of electron‐phonon coupling. In addition, the isotopic distribution of Ta (the element that dominates the acoustic vibration mode carrying heat) is almost entirely composed of the same nuclide, which maximally suppresses the interference of isotopic scattering on acoustic phonons (the main heat carrier). Under the synergistic effect of these triple scattering suppression mechanisms, the predicted room temperature thermal conductivity of θ‐TaN reaches 1000 Wm^−1^K^−1^. In terms of experimental research, Lee et al. [46] and Liu et al. [156] successfully synthesized submicron‐sized θ‐TaN polycrystalline samples using high‐temperature and high‐pressure technology. The measured thermal conductivity (Figure 8f) is one order of magnitude lower than the theoretical value, but still one order of magnitude higher than ε‐TaN [155]. The difference was attributed to multi‐grain boundary scattering and finite‐size effects. Lee et al. [46] considered all phonon scattering mechanisms and calculated the temperature dependence of κ_ph_ under different grain sizes, highly consistent with the experimental results, confirming the ultra‐high thermal conductivity of θ‐TaN.

Subsequently, Li et al. [51] successfully synthesized single‐crystal θ‐TaN and measured the temperature‐dependent thermal conductivity of θ‐TaN in the range of 150–600 K. As shown in Figure 8g, its room‐temperature thermal conductivity reaches as high as 1100 Wm^−1^K^−1^ and decreases with increasing temperature. Electrical measurements of θ‐TaN reveal a high electrical conductivity of approximately 1.5 × 10^6^ S/m. However, according to the Wiedemann–Franz law, the electronic contribution to thermal conduction is negligible, indicating that thermal transport in θ‐TaN is predominantly governed by phonons. As shown in Figure 8h, synchrotron‐based inelastic X‐ray scattering reveals a distinctive phonon band structure with a large acoustic–optical gap and phonon bunching, which suppress phonon–phonon scattering. Furthermore, first‐principles calculations decomposed the phonon scattering rates into four main channels to evaluate different scattering mechanisms (Figure 8i). Three‐phonon processes dominate across most of the phonon spectrum but are significantly suppressed in the high‐frequency acoustic range (6–8 THz) due to the combined effects of the phonon band gap and phonon focusing. This suppression amplifies the relative contribution of higher‐order four‐phonon scattering processes. In contrast, isotopic and electron–phonon scattering remains negligible across the entire phonon spectrum. Finally, the electron–phonon coupling strength was experimentally characterized by measuring the energy relaxation dynamics of hot electrons using ultrafast optical spectroscopy. As shown in Figure 8j, the time evolution of the squared full width at half maximum (Δ(FWHM^2^)) of the spatial distribution of hot electrons is presented for θ‐TaN, Cu, and Al. In Cu and Al, strong electron–phonon coupling results in energy relaxation into the lattice with a characteristic time of only about 1 ps. In contrast, the electron relaxation time in θ‐TaN is measured to be approximately 15 ps—an order of magnitude longer than that in conventional metals. This directly demonstrates the weak electron–phonon interaction in θ‐TaN.

Kundu et al. [157] studied the thermal conductivity of θ‐TaN under high pressure. The pressure‐dependent study (Figure 8k) shows that the thermal conductivity in both directions of TaN increases with increasing pressure, reaching a peak at around 60–80 GPa and then showing a decreasing trend. Their research shows that phonon‐phonon scattering decreases with increasing pressure, especially four‐phonon scattering, which is more sensitive to pressure. In contrast, electron‐phonon scattering increases with increasing pressure. Under the combined effect of these two mechanisms, the thermal conductivity exhibits a non‐monotonic pressure dependence.

Ding et al. [158] theoretically investigated tetragonal TaN (t‐TaN) and discovered a phonon spectrum with a large frequency band gap, similar to θ‐TaN, indicating weak phonon‐isotope and phonon‐phonon scattering. The low density of states near the Fermi level further reduces phonon‐electron scattering. Thus, t‐TaN was expected to be a promising high thermal conductivity semiconductor material. The t‐TaN has a body‐centered tetragonal structure (space group I4_1_md). Due to its phonon properties and bonding heterogeneity, t‐TaN exhibits high thermal conductivity and strong anisotropy, as shown in Figure 8l, with a room temperature thermal conductivity of 677 Wm^−1^K^−1^ along the a‐axis direction and 273 Wm^−1^K^−1^ along the c‐axis direction. At the same time, t‐TaN also exhibits ultra‐high intrinsic hole mobility, with a hole mobility of 4700 cm^2^/Vs along the a‐axis direction, while the hole mobility along the c‐axis is comparable to BAs [160, 161], at 2034 cm^2^/Vs. Overall, this material possesses both high thermal conductivity and high carrier mobility, making it an ideal candidate material for the next generation of electronic and optoelectronic devices.

### 3.4 Application of Machine Learning in Thermal Conductivity Prediction

In recent years, machine learning has emerged as a transformative tool in materials science, offering the capability to overcome the limitations of traditional experimental and computational approaches, thereby accelerating the discovery and optimization of materials [162, 163, 164, 165, 166, 167]. As discussed in the preceding sections, significant progress has been made in elucidating the fundamental thermal transport mechanisms in UHTCs, along with the accumulation of experimental and first‐principles datasets. However, despite these advances, the development of UHTCs remains largely constrained by the high cost and technical difficulty of high‐temperature measurements, as well as the inefficiency of exploring vast compositional and structural spaces using conventional trial‐and‐error strategies or purely first‐principles calculations.

In this context, ML provides a natural bridge between mechanism understanding and materials design. By learning from existing datasets, ML models can predict thermal conductivity with high efficiency, enabling the extraction of structure‐property relationships that are difficult to access through conventional approaches. As a result, ML not only accelerates high‐throughput screening of candidate materials but also serves as a complementary tool for uncovering the underlying thermal transport mechanisms.

**FIGURE 9:**
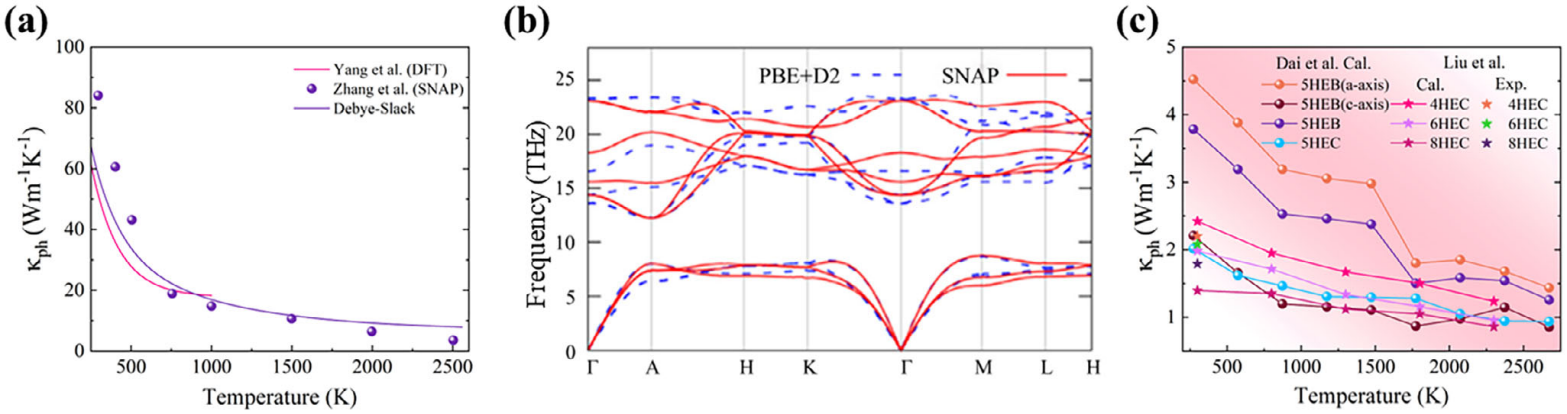
Thermal properties of ultra‐high temperature ceramics (UHTCs) predicted by machine learning (ML). Thermal conductivity (a) and phonon dispersions (b) of ZrB_2_ predicted by ML [168] and its comparison with results from DFT calculations. (c) Comparisons between thermal conductivity predicted by ML and experimental values for high‐entropy ceramics [169, 170, 171]. Reproduced with permission [168]. Copyright 2020, Elsevier.

Recent studies have demonstrated the effectiveness of ML in thermal conductivity research across different material systems, ranging from single‐compound ceramics (ZrB_2_, ZrO_2_) to multi‐component systems (HECs, RE_2_Zr_2_O_7_). These approaches span both ML‐driven interatomic potentials for molecular dynamics simulations and data‐driven regression models for direct property prediction. Beyond achieving efficient and accurate predictions, these studies have revealed intrinsic correlations between composition, crystal structure, and thermal conductivity, thereby providing a new paradigm for mechanism‐informed and performance‐oriented design of UHTCs. Zhang et al. [168] developed a ML‐FF based on the spectral neighbor analysis potential (SNAP), which successfully achieved accurate prediction of thermal properties of ZrB_2_ (including thermal expansion and thermal conductivity) in the ultra‐high temperature range of 300–2500 K. The phonon dispersion relations calculated by this force field are highly consistent with the results of DFT (Figure 9b), verifying the reliability of its description of the structural and dynamic properties of ZrB_2_. Meanwhile, the predicted κ_ph_ in the range of 300–2500 K (results shown in Figure 9a) exhibits excellent consistency with the DFT calculation results by Yang et al. [115]. This work not only provides key data support for the high‐temperature engineering applications of ZrB_2_, but also opens up a new “high accuracy—wide temperature range—high efficiency” path for the research on high‐temperature thermal properties of other UHTCs. Dai et al. [169] developed a deep learning potential and predicted the thermal properties of 5HEC ((Zr_0.2_Hf_0.2_Ti_0.2_Nb_0.2_Ta_0.2_)C) in the temperature range of 300–2700 K via MD simulations. The results showed that the thermal expansion coefficient increases linearly with temperature, while the κ_ph_ decreases from 2.02 to 0.95 Wm^−1^K^−1^ (Figure 9c). This low κ_ph_ originates from lattice distortions caused by differences in the sizes of metal atoms (with carbon atoms deviating more significantly from their ideal positions), providing key insights for analyzing the low thermal conductivity mechanism of HECs. Subsequently, they extended this method to 5HEB ((Ti_0.2_Zr_0.2_Hf_0.2_Nb_0.2_Ta_0.2_)B_2_) [170] and revealed the anisotropy of thermal conductivity in HEBs. The κ_a_ and κ_c_ decreased from 4.55 and 2.22 Wm^−1^K^−1^ to 1.46 and 0.85 Wm^−1^K^−1^ (Figure 9c), respectively. Additionally, the room‐temperature κ_ph_ (∼4 Wm^−1^K^−1^) of 5HEB was higher than that of 5HEC. Liu et al. [171] constructed a transferable neuroevolution potential (NEP) based on a small dataset of unary/binary carbides. They predicted the thermal properties of 4HEC ((Ta_0.25_Nb_0.25_Ti_0.25_Zr_0.25_)C), 6HEC ((Ta_1/6_Nb_1/6_Ti_1/6_Zr_1/6_Hf_1/6_Mo_1/6_)C), and 8HEC ((Ta_1/8_Nb_1/8_Ti_1/8_Zr_1/8_Hf_1/8_Mo_1/8_V_1/8_W_1/8_)C) via MD simulations, and the predicted results showed good agreement with those from first‐principles calculations and experimental measurement data. Within the temperature range of 300–2300 K, the κ_ph_ decreased with increasing temperature; furthermore, the more transition metal elements in HECs, the more pronounced the reduction in κ_ph_ (with the κ_ph_ of 8HEC being 30% lower than that of 4HEC). Additionally, they confirmed that strain fluctuations, rather than mass fluctuations, were the main cause of the low thermal conductivity.

These results demonstrate that ML can achieve predictive accuracy comparable to ab initio methods while significantly reducing computational cost, enabling high‐throughput screening of complex UHTC systems and providing insights into phonon transport and structure‐property relationships. However, its application remains limited by the scarcity and heterogeneity of high‐quality data, as thermal transport depends on coupled effects of composition, microstructure, and synthesis conditions, while existing datasets are often sparse and inconsistent, and first‐principles calculations remain expensive for multicomponent systems. Addressing these challenges will require standardized data frameworks, integration of experimental and computational datasets, and data‐efficient modeling strategies such as physics‐informed and multi‐fidelity learning, together with closed‐loop approaches that couple ML predictions with targeted experiments, ultimately enabling the accelerated design of UHTCs with tailored thermal properties.

### 3.5 Micro/Nano Structural Engineering of Thermal Conductivity

The optimization of thermal transport performance of UHTCs provides key support for their reliable service in extreme environments and further expands their application boundaries. Multiple thermal conductivity control strategies have been developed, including porous, high‐vacancy UHTCs and composite materials.

**FIGURE 10:**
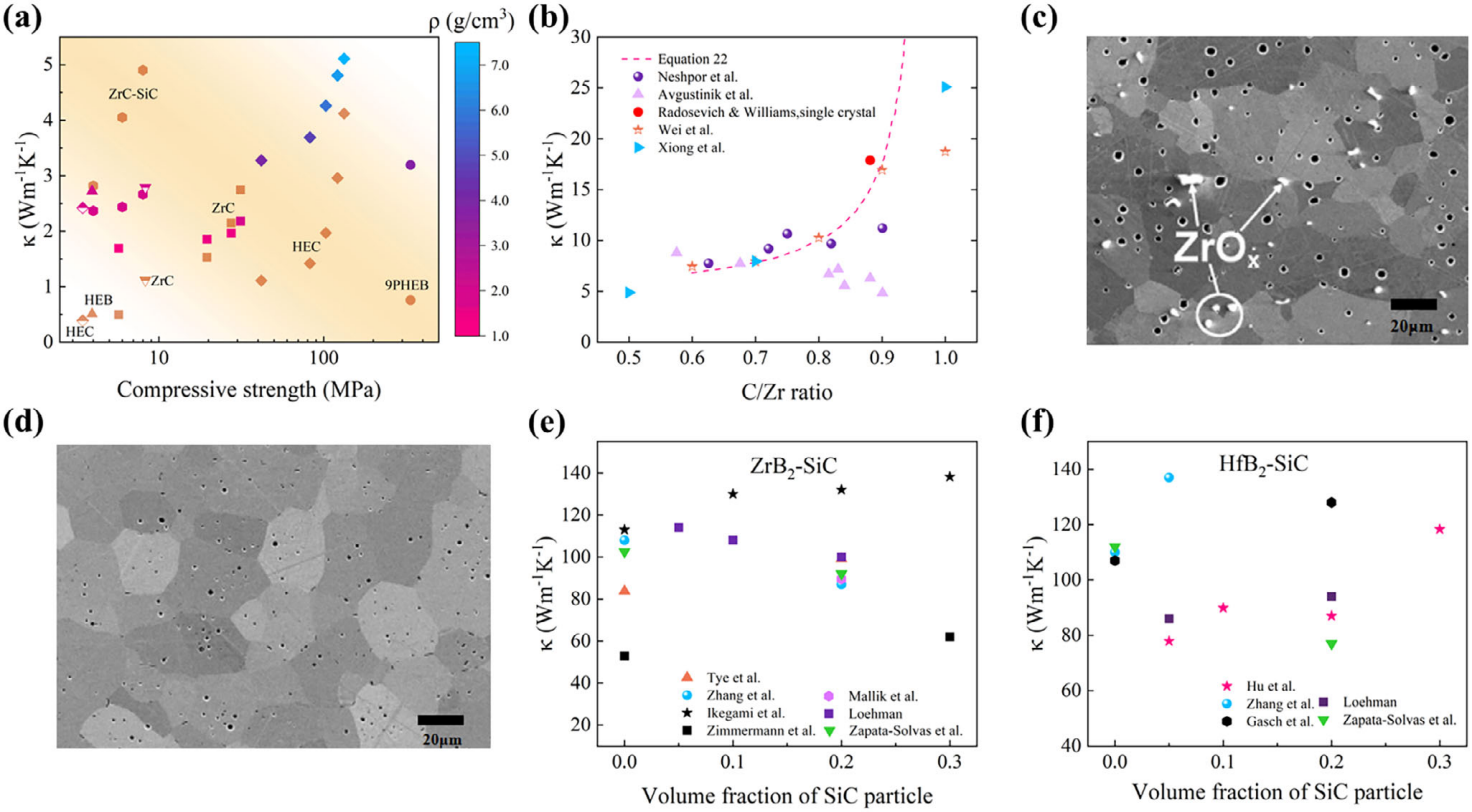
Control of thermal conductivity of ultra‐high temperature ceramics (UHTCs). (a) Porous ultra‐high temperature ceramics with low thermal conductivity, low density, and moderate compressive strength [172, 173, 175, 176, 177, 178, 179]. (b) Results of total thermal conductivity of ZrC as a function of different carbon zirconium ratios [24, 140, 181, 182]. SEM images of (c) ZrB_2_ and (d) ZrB_2_ with 2wt.% carbon added. Reproduced with permission [104]. Copyright 2015, Elsevier. The addition of carbon can remove oxygen‐containing phases and improve the degree of densification. Thermal properties of ZrB_2_‐SiC [36, 37, 117, 130, 131, 191] (e) and HfB_2_‐SiC [30, 37, 130, 131, 192] (f).

Porous UHTCs exhibit significant advantages due to their unique multi‐level pore structure design. By enhancing the phonon‐defect scattering, these materials significantly reduce their thermal conductivity while possessing multiple properties such as low density, low thermal conductivity, high porosity, and high sound absorption coefficient. In addition, they can maintain excellent structural stability over a wide temperature range, a core advantage that makes them ideal materials for efficient thermal insulation and acoustic control in extreme environments. Wen et al. [172] fabricated (Hf_1/9_Zr_1/9_Ta_1/9_Nb_1/9_Ti_1/9_Cr_1/9_Mo_1/9_V_1/9_W_1/9_)B_2_ (9PHEB) high‐entropy ceramics through multi‐scale structural design. Studies revealed that this material maintains high stability even at 2000°C, and retains a compressive strength of 337 MPa at a porosity of 50%, with a thermal conductivity as low as 0.76 Wm^−1^K^−1^. Yang et al. [173] fabricated (Ta_0.2_Nb_0.2_Ti_0.2_Zr_0.2_Hf_0.2_)C dual‐porosity high‐entropy ceramics via pressureless sintering combined with the carbothermal reduction method. The small pores (0.4–3 µm) originate from the carbothermal reduction of the ceramic precursor, while the large pores (20–50 µm) are introduced by SiO_2_ microspheres. This ceramic exhibits a thermal conductivity of 1.11–4.12 Wm^−1^K^−1^ and a compressive strength of 41.9–133.1 MPa. Li et al. [174] prepared highly porous ZrC by the foaming method, and obtained zirconium carbide with a porosity of 85%, a uniform pore structure with an average size of about 40 microns, and an extremely low thermal conductivity value (0.94 Wm^−1^K^−1^) and excellent high‐temperature stability. However, the compressive strength at room temperature is only 0.4 MPa, and further thermal mechanical adjustment is still needed to obtain materials suitable for application. Chen et al. [175] prepared high porosity, low thermal conductivity ZrC and HfC materials through an in situ reaction/partial sintering process. The porosity of ZrC reaches 68.74%, with a density of 2.06 g/cm^3^, which is 31.2% of the theoretical density. The thermal conductivity at room temperature is 1.12 Wm^−1^K^−1^, and the compressive strength is 8.82 MPa. The porosity of HfC is as high as 77.82%, with a density of 2.82 g/cm^3^, which is only 22% of the theoretical density. The thermal conductivity at room temperature is 1.01 Wm^−1^K^−1^, and the compressive strength is 5.51 MPa. The team further introduced the design concept of high entropy ceramics into porous systems [176], and developed (Zr, Hf, Ta, Nb)C high entropy ceramics with a porosity of 80.99%, a density as low as 1.79 g/cm^3^, and a compressive strength of 3.45 MPa, while maintaining ultra‐low thermal conductivity of 0.39 Wm^−1^K^−1^. The porous high entropy ceramics can be stably maintained at least up to 1850°C in an argon atmosphere. Yan et al. [177] used a combination of solvent evaporation and hot pressing sintering to prepare porous ZrC ceramics with controllable porosity and pore size. The density of ZrC with 73.5% pores is only 1.25 g/cm^3^. This high porosity can significantly reduce thermal conductivity by affecting phonon transport, with a thermal conductivity of only 0.5 Wm^−1^K^−1^ below 1000°C. The sound absorption coefficient of the material is higher than 0.5 in the frequency range of 2.1–3.4 kHz, with a high absorption coefficient of 0.96 at 2.7 kHz. At the same time, Yan et al. also evaluated the mechanical properties of porous ZrC and found that the compressive strength of ZrC with different pores ranged from 5.7 to 31 MPa, demonstrating a good ability to control compressive strength porosity. Wang et al. [178] fabricated porous ZrC‐SiC ceramics from zircon (ZrSiO_4_) and carbon black via a one‐step sintering technique, which combines in situ carbothermal reduction and partial hot‐pressing sintering. The ceramics exhibit a porosity of 61.37%–70.78%, compressive strength of 1.31‐7.48 MPa, and room‐temperature thermal conductivity of 1.48–4.90 Wm^−1^K^−1^. Their pore structure and properties can be tuned by adjusting sintering pressure, making them potential lightweight high‐temperature thermal insulation materials. Figure 10a [172, 173, 175, 176, 177, 178, 179] summarizes the performance of porous UHTCs in recent years. The thermal conductivity and density of the material are negatively correlated with the compressive strength of the pores. How to coordinate the thermal‐mechanical balance to achieve the best performance is an important issue.

ZrC_x_ sub‐stoichiometric ceramics based on carbon vacancy regulation exhibit unique advantages in the fields of nuclear energy and high‐temperature insulation. According to the Zr‐C phase diagram [180], ZrC_x_ can remain stable when the C/Zr molar ratio (x value) is in the range of 0.6–1.0. This non‐stoichiometric characteristic endows the material with special adjustable properties: Wei et al. [140] prepared ZrC_x_ (x ranging from 0.6 to 1) using ZrC and ZrH_2_ powders through two‐step reaction hot pressing, and studied its densification, mechanical, and thermal properties. Research has found that when x < 1, the degree of densification increases (> 96%), while the final densification degree of ZrC with x = 1 is 91.9%. And as the C/Zr ratio decreases, the mechanical properties also decrease. The elastic modulus decreased from 390 GPa at x = 0.9 to 260 GPa at x = 0.6. The bending strength is greatly affected by size, but for ZrC_x_ with different C/Zr ratios that are close in size, the change in bending strength is not significant (320 MPa). The hardness also did not change significantly and was affected by pores. The hardness of ZrC_0.6_ was even higher than that of ZrC with a low densification degree. Xiong et al. [24] studied the mechanical and thermal properties of densified ZrC_x_ (x = 0.5, 0.7, 1), and obtained highly densified samples (> 98%) through spark plasma sintering and vacuum arc melting methods. The mechanical properties decrease with the decrease of x. The Vickers hardness decreases from 28.8 GPa at x = 1 to 20 GPa at x = 0.5, and the Young's modulus decreases from 450 GPa at x = 1 to 390 GPa at x = 0.5. And the thermal conductivity decreased significantly, from 25 Wm^−1^K^−1^ at x = 1 to 4.9 Wm^−1^K^−1^ at x = 0.5 at room temperature. Figure 10b shows the κ of ZrC at room temperature under different C/Zr ratios. The thermal conductivity decreases with the decrease of the carbon zirconium ratio, and carbon vacancies lead to an increase in phonon and electron scattering. Storms [181] proposed an empirical equation (Equation 22) to describe the variation of ZrC thermal conductivity with C/Zr ratio at room temperature:

(22) $$\begin{array}{ccc} k & = & 1.05\times 103\;\left(0.00382+\frac{1}{55+950\times \left(1-x\right)}\right)\\ & & +\;\frac{0.07}{{\left(1-x\right)}^{2}}\left(0.6\leq x\leq 1\right) \end{array}$$

which is also shown in Figure 10b (red dashed line). Except for the deviation between Avgustinik et al.’s [182] results and Equation 22, all other results are relatively close. The carbon vacancies in ZrC_x_ can serve as absorption points for interstitial atoms generated by irradiation, improving its tolerance to radiation damage [183, 184]. Therefore, it has great potential in accident‐tolerant fuels and nuclear fuels. And this introduction of vacancies to reduce thermal conductivity without causing significant changes in mechanical properties is of great significance for ZrC in the field of high‐temperature insulation.

Fiber composite UHTCs are composite materials formed by combining carbon fibers with UHTCs. Through material design and process optimization, they demonstrate unique advantages in extreme high temperature environments [185, 186, 187]. Specifically, it is manifested as a significant improvement in fracture toughness; low density; optimization of thermal seismic performance; anisotropic regulation of thermal conductivity; optimization of anti‐oxidation and erosion resistance performance, etc. Yu et al. [188] employed graphene fibers as the substrate and successfully fabricated graphene/titanium carbide composite fibers (GTF) with a well‐defined core–shell structure via a one‐step molten salt synthesis method, achieving both high thermal conductivity and excellent structural stability. By adjusting the sintering temperature and duration, they systematically tuned the grain size and shell thickness of the TiC coating. The nanoscale TiC layer introduced additional phonon scattering channels, which somewhat reduced thermal conductivity, while a thicker coating contributed to improved thermal shock resistance. With an optimized shell thickness of 1 µm, the single GTF exhibited a thermal conductivity of 745 Wm^−1^K^−1^, along with outstanding thermal shock resistance and no interfacial failure, ensuring its long‐term durability under extreme conditions. Furthermore, the GTF woven fabric demonstrated exceptional ablation resistance when subjected to an oxyhydrogen flame at 2200°C, with a mass ablation rate as low as 0.3 mg/s. Unlike the fiber‐reinforcement strategy discussed above, oxide dispersion strengthening via incorporating nanoscale dispersoids into metallic matrices is also a critical approach for improving ultrahigh‐temperature load‐bearing capacity. Xue et al. [189] employed a Ta‐12W‐1Re base alloy with a small addition of HfB_2_ (0.4 wt.%) and utilized the inevitable oxygen impurities in the matrix to generate HfO_2_ nanoparticles via a boron‐intervened in situ oxidation reaction. The resulting oxide particles, with an average diameter of about 50 nm, are uniformly dispersed within the grain interior, while boron atoms segregate at the particle/matrix interfaces and grain boundaries, suppressing particle coarsening and preventing preferential grain‐boundary precipitation. After thermomechanical processing and recrystallization annealing, the alloy exhibits a uniform equiaxed grain structure with an average grain size of approximately 14 µm. Mechanically, it achieves a room‐temperature yield strength of 720 MPa, an ultimate tensile strength of 830 MPa, and an elongation‐to‐failure of 35%, combining high strength with excellent ductility. At elevated temperatures, the tensile yield strength reaches about 200 MPa at 2000°C and remains about 100 MPa even at 2400°C, substantially outperforming all conventional refractory alloys and refractory multi‐principal‐element alloys. These studies collectively highlight the great potential of micro/nano engineering in optimizing the comprehensive performance of materials under extreme thermo‐mechanical coupling environments, and offer new design strategies for future ultrahigh‐temperature composites aimed at ever more demanding service conditions.

The diffusion barrier formed by residual oxides (such as ZrO_2_) on the surface of particles during the densification process of UHTCs is a key factor restricting sintering efficiency. By introducing reducing additives such as carbon/boron carbide, oxidation‐reduction reactions can occur with surface oxides, effectively eliminating oxygen impurities and promoting the densification process. Thompson et al. [102] studied the effect of adding 0%–3 wt% C during the sintering of ZrB_2_ and found that the addition of carbon can accelerate the time required for densification. ZrB_2_ without carbon addition takes 35 min to reach near complete densification at 1900°C, while samples with 1–3 wt.% C can be densified in only 10 min. The addition of carbon reduces the oxygen content and size of the sample. The grain size of ZrB_2_ is reduced through two different mechanisms: (1) Carbon addition shortens the time required for densification, thereby reducing grain coarsening; (2) The addition of carbon to generate a second phase leads to grain fixation. Harrington et al. [104] and Zhou et al. [118] also reached similar conclusions, and the degree of densification increased with the increase of carbon content. As shown in Figure 10c,d, the addition of 2 wt.% carbon can eliminate oxygen‐containing phases, thereby improving the degree of densification. Boron carbide, as an additive, has the same effect of promoting densification by inhibiting oxidation. Zhang et al. [190] studied the effect of boron carbide on the pressureless sintering of ZrB_2_, and the results showed that ZrB_2_ containing only WC could be sintered to 95% relative density within 4 h under vacuum conditions at 2050°C. After adding boron carbide, it can be sintered to a relative density greater than 98% within 1 h under vacuum conditions at 1850°C. Boron carbide, as a sintering aid for ZrB_2_, can react with zirconia to form ZrB_2_ and promote its densification at lower temperatures. Neuman et al. [112] investigated the high‐temperature thermal properties of ZrB_2_‐B_4_C ceramics. With the addition of boron carbide, the grain size decreased significantly, from 22 µm in pure ZrB_2_ to 5.4 µm with the addition of 15 vol.% boron carbide. However, the addition of boron carbide also reduces the thermal conductivity of ZrB_2_, with a decrease of approximately 15% at 300 K (from 93 to 79 Wm^−1^K^−1^). Current research reveals that by precisely regulating the types and contents of additives, the sintering process of UHTCs can be optimized while maintaining high thermal conductivity, saving costs, and providing a new paradigm for low‐cost manufacturing of UHTCs.

UHTC and silicon carbide (SiC) composites have been studied extensively. ZrB_2_ composites with SiC addition have improved the degree of densification as well as mechanical properties, oxidation resistance, and thermal properties. The thermal properties of UHTCs and silicon carbide composites are regulated with complex characteristics. Taking ZrB_2_‐SiC and HfB_2_‐SiC systems as examples (Figure 10e,f), although single‐crystal SiC has an intrinsic thermal conductivity of up to 490 Wm^−1^K^−1^ [193], the thermal transport capacity of the SiC phase in actual composite systems is significantly affected by the preparation process. The thermal conductivity of SiC varies in gradient with the difference of sintering additives: different sintering agents change the thermal conductivity of SiC from 80 to 270 Wm^−1^K^−1^ [194, 195, 196]. This indicates that the purity and defect state of the SiC phase in composites play a decisive role in heat transport. Therefore, SiC should be a high thermal conductivity phase with respect to ZrB_2_ and HfB_2_, and the addition of SiC was tentatively predicted to increase the thermal conductivity. Different from UHTCs, SiC is an insulator, and its high thermal conductivity is due to the contribution of phonons, but the increase of impurity content will greatly limit the phonon transport, which makes the thermal conductivity of SiC decrease sharply. For example, the room temperature thermal conductivity can be reduced to 60 Wm^−1^K^−1^ when the SiC contains N impurities [197], almost a magnitude lower than that of the single crystal. Thus, for composites, the mass of SiC has a large effect on the thermal conductivity. ZrB_2_‐SiC composites prepared by Zhang et al. [37], Zapata–Solvas et al. [131], and Loehman et al. [130], show that the addition of SiC results in a reduction in thermal conductivity (Figure 10e), which may be due to the introduction of impurities during the preparation, reducing the quality of SiC. Most reported room temperature thermal conductivity is between 80 and 100 Wm^−1^K^−1^ when the content of SiC in the composite is 20 vol.%. Ikegami et al. [117] reported the highest thermal conductivity, with a lower ZrB_2_ density (95.6%) and an unusually high heat capacity, which was significantly reduced (93 Wm^−1^K^−1^) after correction according to the Maxwell Ecken relation. Although their composites are highly dense, the measured heat capacity also has the potential for large errors, assuming consistent with the drop in room temperature thermal conductivity, the thermal conductivity of the 20 vol.% SiC content is close to most reports. Zimmermann et al. [36] report the lowest value of thermal conductivity, employing ball milling of the WC medium, and W reduces the thermal conductivity of ZrB_2_, as mentioned earlier. Overall, the composite thermal conductivity of ZrB_2_ SiC has good agreement. As for HfB_2_‐SiC composites, in contrast, only the results of Zapata–Solvas et al. [131] show that the thermal conductivity of HfB_2_‐SiC composites decreases with the addition of SiC, and the rest of the studies show an upward trend. While three of these have higher thermal conductivity values, around 120 Wm^−1^K^−1^, the 5 vol.% SiC samples reported by Zhang et al. [37] reach 137 Wm^−1^K^−1^, and the 20 vol.% samples reported by Gasch et al. [30] reach 130 Wm^−1^K^−1^, possibly due to the higher mass of its own HfB_2_, and the addition of SiC may trap impurities, resulting in high thermal conductivity. For composites at high temperatures, the thermal conductivity of HfB_2_ decreases from 112 to 100 Wm^−1^K^−1^ from room temperature to 700 K, and HfB_2_ with 5% SiC reduces from 137 to 110 Wm^−1^K^−1^ [37]. Loehman et al. [130] report even two opposite results, with the addition of SiC, thermal conductivity is increased at room temperature for the composites, but the value decreases at high temperatures. Other research groups have reached similar conclusions at high temperatures [30, 36]. The reason for this is that the phonon scattering is increased by temperature, and the thermal conductivity of SiC is greatly reduced, and the thermal conductivity of composite materials is more affected by temperature than that of pure UHTCs, resulting in a more decrease trend. Finally, because there are many factors affecting the thermal conductivity of composites, such as the mass of the material itself and the mass of SiC, there is no obvious dependence of the thermal conductivity on the content of SiC. Although the introduction of SiC does not cause drastic fluctuations in the thermal conductivity, fine regulation of the thermal transport properties of composites can be achieved by regulating the mass of the second phase and the preparation process. This property provides an important regulatory dimension for the thermal management design of ultra‐high temperature components.

The discussion above indicates that micro/nano‐structure engineering is not an isolated means of structural modification, but rather a central regulatory strategy that directly influences the thermoelectric transport process. By introducing pores, vacancies, secondary phases, and dislocations, structural engineering can significantly enhance the scattering spectrum of phonons, thereby substantially reducing lattice thermal conductivity—a key pathway to improving the thermoelectric figure of merit (ZT). At the same time, appropriate band engineering and interface design can enhance the Seebeck coefficient while maintaining high electrical conductivity, thereby optimizing the power factor. This will be discussed in detail later in the context of thermoelectric performance optimization. Therefore, micro/nano‐structure engineering essentially provides a physical mechanism foundation for thermoelectric performance optimization. Building upon this mechanism, researchers are able to achieve the ideal “phonon‐glass electron‐crystal” transport characteristics in material systems, driving sustained breakthroughs in thermoelectric performance.

## 4 Thermal Radiation Characteristics and Management

Actually, a complete UHTCs thermal system involves a continuous and coupled process encompassing conductive, convective, and radiative processes. However, at low to moderate temperatures, radiative heat transfer is often negligible compared to conduction. While unlike heat conduction and convection, which scale linearly with temperature or temperature gradient, the rate of thermal radiation increases with the fourth power of temperature according to the Stefan–Boltzmann law [86]. As a result, thermal radiation can dominate over conduction and convection at sufficiently high temperatures and become a quite important factor to consider. One of the widely studied applications of UHTCs’ radiative properties is in thermal protection systems [198]. When hypersonic vehicles fly at speeds exceeding Mach 5, intense heating is generated at the leading edge. To prevent structural failure from extreme thermal loads, UHTC‐based coatings are often employed as thermal shields, where the low thermal conductivity provides effective insulation by impeding heat penetration from the external high‐temperature environment, and thermal radiation enables the generated energy to be dissipated from the surface. Because thermal radiation is the dominant cooling mechanism at these temperatures, enhancing the infrared emissivity of UHTCs coatings is a key strategy for TPS design—as exemplified by the passive system shown in Figure 1d. Another typical application of UHTCs is in concentrating solar power systems [199], where solar absorbers are exposed to concentrated solar radiation, leading to elevated surface temperatures and intense near‐infrared thermal radiation. The extreme condition demands materials that not only withstand extreme heat but also exhibit favorable optical properties, such as high solar absorptance and low infrared emissivity, to ensure high photothermal conversion efficiency.

In recent years, extensive research efforts have focused on understanding and tailoring the radiative behavior of UHTCs for these high‐temperature applications. This section introduces the intrinsic radiative properties of UHTCs and the main strategies and recent progress reported in the literature for modifying the radiative and optical parameters of UHTCs, including both IR emissivity enhancement and solar spectral selective absorption.

### 4.1 Intrinsic Radiative Properties of UHTCs

According to Planck's law, objects at 1000–3000 K emit thermal radiation predominantly in the infrared wavelength range. Thus, the emissivity of UHTCs within the wavelength range will significantly influence the application of the material.

Borides count as an important part of UHTCs, and they often have low intrinsic IR emissivity. Sani et al. [200] reported their study on the optical characterization of ZrB_2_ and HfB_2_ dense samples. The total hemispherical emissivity was measured at 1100–1700 K, and the value ranges from 0.35 to 0.45 (Table 2). Also, the spectral research revealed that the main contribution to the emittance is given by radiation at a wavelength range shorter than 2.8 µm. Zhang et al. [201] studied the infrared properties of sputter‐deposited post‐annealed ZrB_2_ films. The low emissivity of ∼0.01 (8–14 µm) and ∼0.05 (3–5 µm) means the film can be used as infrared stealth film at high temperature environment. Recently, Su et al. [202] measured the IR emissivity of ZrB_2_ thin films fabricated using magnetron sputtering technology and showed that the emissivity of the film can stabilize at 0.2 and 0.1 at the wavelength range of 3–5 µm and 8–14 µm, respectively, further confirming the material's low IR emissivity potential for application in infrared stealth. Tu et al. [203] comprehensively analyzed the low infrared emissivity of HfB_2_ from the aspect of electronic structure. Their first principles analysis results reveal that the light reflection properties of HfB_2_ material in the infrared region are mainly governed by two key factors: plasma frequency and damping factor, which indicates that adjusting the free electron concentration and electron scattering can be effective methods to tailor the emissivity of this material. They then tried to increase the thickness of the film and roughen the substrate to further decrease the emissivity. The IR emissivity is reported below 0.28 through the experiment, a suitable feature for stealth.

**TABLE 2:** The thermal radiation emissivity of several typical ultra‐high temperature ceramics [200, 201, 202, 203, 204, 205, 206, 207, 208, 209, 210, 211, 212].

Material	Fabrication method	Test temperature	Wavelength range, µm	ε_em_
ZrB_2_	dense sample	∼1150K	total	∼0.35
		∼1430K	total	∼0.45
	sputter‐deposited		3–5	0.05
			8–14	0.01
	magnetron sputtering		3–5	0.20
			8–14	0.10
HfB_2_	dense sample	∼1300K	total	∼0.40
		∼1690K	total	∼0.45
	sputter‐deposited		3–5	0.23
			8–14	0.14
ZrC	coating	1700–2200 K	0.81	0.90
	spark plasma sintering		0.4–0.9	∼0.60
HfC	coating	1700–2200 K	0.81	0.90
HfC_0.91_	pulsed DC magnetron co‐sputtering		8–14	0.10
TaC	coating	1800–2400 K	0.81	0.43
NbC	coating	1700–2200 K	0.81	0.79
TiN_x_	sputter‐deposited (80 W)		8–14	0.10
TiN	sputter‐deposited (130 W)		8–14	∼0.26
TiCrN	magnetron co‐sputtering		8–14	∼0.30

Comparatively, carbide UHTCs have higher IR emissivity. Ozaki et al. [205] measured the emissivity at 0.81 µm of several carbides for the application in systems that acquire effective radiative coupling, like a propulsion system with nuclear fuel. As shown in Table 2, the components with ZrC and HfC coatings exhibit a high emissivity of 0.9 at 1700–2200 K, while those with TaC and NbC coatings have a relatively low emissivity of 0.43 at 1800–2400 K and 0.79 at 1700–2200 K, respectively. Besides, the long‐term stability of ZrC and HfC further proves the materials are suitable for advanced nuclear energy conversion applications. Manara et al. [207] reported the spark‐plasma‐sintered ZrC sample's emissivity about 0.6 at the wavelength range of 0.4–0.9 µm, consistent with the previous research data in general. However, through adjusting HfC's C stoichiometry and energy input when produced, Zou et al. [210] construct a HfC_0.91_ film which can reach a quite low emissivity of 0.1 at 8–14 µm as a result of the synergistic effect of reduced lattice vibration absorption, reduced plasma energy and enhanced multiple scattering absorption. Together with the property of low average visible reflectivity of 0.33, it also presents promising potential for application in actual multispectral camouflage systems designed for harsh environments. Also, the potential ideal spectral selectivity and extremely high working temperature make the carbide UHTCs promising candidates for present SiC or metallic solar absorbers [89].

Nitrides have similar emissive characteristics to borides. For example, Xu et al. [204] explored the specific IR radiation properties of the sputtering‐deposited TiN_x_ film and the influence of sputtering power (50, 80, 100, and 200 W) on the IR emissivity (8–14 µm). As the sputtering power increases from 50 to 200 W, the emissivity of TiN_x_ films drops from 0.23 to 0.10 and then rises to 0.13. It is explained that nitrogen ion vacancies caused by the deficit in nitrogen atoms in the TiN_x_ lattice result in the release of correlative electrons as the ratio of N/Ti is less than 1 at 80–200 W. These electrons are bound around the nitrogen vacancies to keep the charge neutral and can be migrated as carriers when they are excited, thus having a reflection effect on the infrared electromagnetic wave and then lowering the emissivity. In the research of Li et al. [208] a similar emissivity of around 0.26 at 8–14 µm is also reported when the film thickness is 645 nm. Xu et al. [209] also researched the infrared emissivity of nonstoichiometric TiCrN films produced by adjusting the nitrogen flux of reactive magnetron co‐sputtering. The lowest emissivity of ∼0.3 is reported when the nitrogen flux is 6 sccm. The IR emissivity can also be explained by the different density of electrons resulting from different stoichiometric ratios of nitrogen.

### 4.2 Dopant‐Induced Emissivity Enhancement

Although some UHTCs exhibit weak intrinsic emissivity and absorption within the infrared wavelength range, doping has been demonstrated as an effective way to enhance the spectral emissivity [213]. Several mechanisms have been proposed to explain doping‐enhanced emissivity: free carrier absorption (an electron within a band absorbs radiation by transferring from a low energy level to an empty high‐energy level), d‐d transitions in octahedral coordination (the absorption of energy as electrons in transition metal ions jump between different energy levels split by the crystal field of an octahedral structure) and lattice distortion (the absorption properties are modified due to the introduction of new energy states or defect levels caused by structural deformations).

SiC is an important secondary phase in ZrB_2_–SiC, improving not only oxidation resistance and mechanical strength but also the radiative properties [198]. SiC exhibits intrinsically high emissivity due to the free‐carrier absorption mechanism in the near‐IR region at high temperatures, making it an efficient additive for enhancing UHTC emissivity [214]. Scatteia et al. [206] investigated the radiative properties of the sintered ZrB_2_‐SiC ceramic composite at high temperatures ranging 1000–1800 K. The total hemispherical emissivity and spectral emissivity at 5 µm in air at 200 Pa and 10^−3^ Pa are measured. The relatively high emissivity of ∼0.75 proves the compound potentially suitable for the manufacturing of thermal protection systems like sharp‐shaped leading edges for the good radiation cooling effect.

**FIGURE 11:**
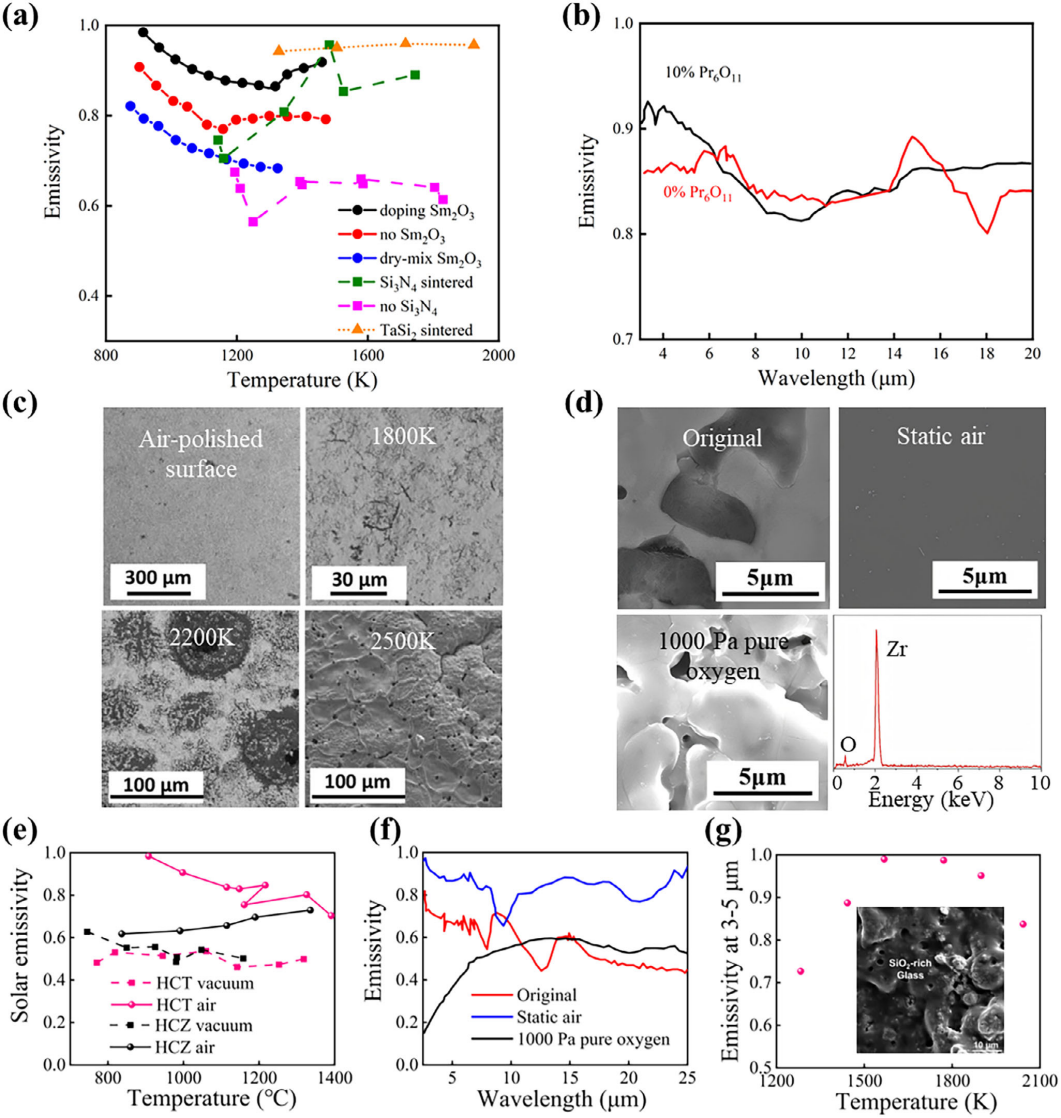
Thermal emissivity and surface characteristics of modified ultra‐high temperature ceramics (UHTCs). (a) Total hemispherical emissivity of ZrB_2_‐SiC modified with different dopants at different temperatures [215, 218, 219]. (b) Spectral emissivity of the Pr_6_O_11_ modified ZrB_2_‐SiC under 3–20 µm band [43]. (c) FE‐SEM images of HCT samples: polished surface, and surfaces after oxidation at 1800, 2200, and 2500 K for 20 min. Reproduced with permission [89]. Copyright 2017, Elsevier. (d) Micrographs and EDS analysis of the surface for ZrB_2_‐SiC specimens. Reproduced with permission [220]. Copyright 2017, Elsevier. (e) Solar emissivity of the HfC‐based ceramics sintered with TaSi_2_ or ZrSi_2_ measured in air and vacuum [89]. (f) Spectral emissivity of ZrB_2_‐SiC specimens pre‐oxidized in different environments as a function of wavelength [220]. (g) Emissivity of ZrB_2_‐SiC as a function of oxidation temperature measured in the 1 MW plasma wind tunnel with the typical surface microstructure. Reproduced under the terms of the CC BY license [221]. Copyright 2021, Liu et al.

Rare‐earth oxides (REOs) and rare‐earth (RE) ions are known for their intrinsically high emissivity in the IR spectral region (0.76–6 µm) at high temperatures. Also, REOs often have melting temperatures exceeding 2473 K and high thermal conductivity, making them well‐suited as functional additives in UHTCs. Extensive research has been conducted to enhance the emissivity of UHTCs, mainly ZrB_2_‐SiC, through doping REOs or RE ions. Tan et al. [215] introduced Sm_2_O_3_ into the ZrB_2_‐SiC using both dry‐mixing and chemical‐doping, respectively. Chemically doped coating has exhibited a distinctly higher total hemispherical emissivity compared to the non‐doping or dry‐mixing ZrB_2_‐SiC. As shown in Figure 11a, the emissivity reaches a local minimum of ∼0.87 at 1050°C and increases to ∼0.93 at 1200°C. Several other groups also investigated the radiative properties of Sm_2_O_3_ modified ZrB_2_‐SiC material [216, 217]. For instance, Xu et al. [216], reported a high emissivity of 0.85 in the 2.5–5 µm band at 1000°C and explained the enhancement mechanism. The oxygen vacancies introduced by Sm_2_O_3_ produce nonstoichiometric point defects, which will introduce intermediate energy levels between the conduction band and the valence band. These defects, along with the electronic configuration of Sm^3+^, localize f‐electronic states and increase the transition of electrons, thus improving the absorption efficiency and accordingly improving the emissivity. The research also noted a significant reduction in surface temperature and better anti‐ablation performance during the ablation tests, as a result of the improved radiative heat dissipation and the formed dense surface structure in a high‐temperature environment. Pr_6_O_11_, another REO with a melting point of 2315 K, also exhibits strong infrared emission centered around 1 µm. Recently, Liu et al. [43] enhanced the radiation properties of ZrB_2_‐SiC coatings by incorporating 10 wt.% Pr_6_O_11_, forming the ZSP10 composition. As shown in Figure 11b, ZSP10 exhibited significantly enhanced IR emissivity. This enhancement can be attributed to the intrinsic emissivity of Pr_6_O_11_ and the localized electronic states in the material's bandgap. The 4f^2^ configuration of Pr^3+^ and Pr^4+^ ions supports strong electronic transitions in the near‐IR, while the oxygen vacancies from non‐stoichiometric Pr_6_O_11_ create localized electronic states in the bandgap, further boosting absorption. In ablation tests, the surface temperature of ZSP10 decreases by 150 K to 1923 K due to its high emissivity.

In addition to the above‐mentioned additives, many other dopants have also been reported to enhance emissivity at elevated temperatures, as shown in Figure 11a. Balat et al. [218] incorporated Si_3_N_4_ as a sintering additive into ZrB_2_‐SiC material. Si_3_N_4_ has a relatively higher emissivity, and a higher concentration of SiO_2_ was observed on the microstructure of the modified samples when oxidized, which accounts for the higher emissivity than the non‐doping samples at the temperature range of 1150–1750 K, proving Si_3_N_4_ an effective kind of additive. Also, Pellegrini et al. [219] introduced replacing SiC with TaSi_2_ when sintering ZrB_2_ and the emissivity value of 0.80 was given at the temperature up to 1977°C. Overall, dopant engineering has proven highly effective in simultaneously improving emissivity, ablation resistance, and surface cooling performance.

### 4.3 Oxidation‐Driven Evolution of Emissivity in UHTCs

In high‐temperature and oxygen‐containing environments, oxidation of UHTCs materials is inevitable and strongly influences radiative properties by altering surface roughness, composition, texture, and so on. Heating tests in an oxygen environment reveal the formation of multiple oxides, with microstructural changes becoming pronounced at higher temperatures [89]. Especially, the common silicon‐supplying additives in UHTCs will produce high‐IR‐emissivity silica after oxidation, and the emergence and dissipation of SiO_2_ will influence the surface radiative properties during high‐temperature exposure [221].

As mentioned above, Scatteia et al. [206] first reported the maximum total hemispherical emittance of the ZrB_2_‐SiC material as 0.77 at 764°C in high temperature oxidizing environment. An in situ glassy borosilicate layer was formed when tested at temperatures above ∼700°C in 200 Pa air pressure, explaining the increased surface emissivity. Charpentier et al. [89] also studied the effect of high temperature oxidation on the radiative properties of HfC‐based ceramics with additives of 10 vol.% TaSi_2_ or ZrSi_2_, named HCT and HCZ, respectively. As shown in Figure 11e, both materials exhibited stable emissivity (∼0.4) in vacuum. However, in the air, emissivity increased sharply, reaching ∼0.95 across the infrared range. Post‐test analysis (Figure 11c) revealed significant oxide growth and microstructural roughening. The enhanced emissivity was attributed to both increased surface roughness after oxidation and the formation of silica, which has a high intrinsic infrared emissivity. To explore the possible application of the oxidation‐induced high emissivity, Li et al. [220] pre‐oxidized ZrB_2_‐20 vol.% SiC composites. The emissivity of the samples pre‐oxidized in static air is higher than that of untreated ZrB_2_‐SiC specimens in the 2.5–25 µm range at 300–900°C as shown in Figure 11f. This improvement was linked to the formation of a dark, glassy borosilicate surface layer (Figure 11d). In contrast, samples oxidized at 1000 Pa in pure oxygen developed a ZrO_2_‐abundant porous, rough surface with poorer radiative properties (Figure 11d). These results confirm that pre‐oxidation is an effective way to improve the UHTCs’ radiative properties for TPS applications, while also indicating that the ZrB_2_‐SiC material should be avoided in low‐pressure pure oxygen environments.

To further understand the influence of oxidation on the radiative properties under flight‐like conditions, Liu et al. [221] systematically measured the emissivity of three typical SiC‐based TPS materials in a 1 MW inductively coupled plasma wind tunnel. Typically for ZrB_2_‐SiC, the results in Figure 11g show that its emissivity is highly dependent on the temperature‐driven oxidation and volatilization of oxides. At 1009–1298°C, oxidation of ZrB_2_ and SiC produced B_2_O_3_ and SiO_2_, raising emissivity from 0.73 to 0.98. Between 1298 and 1497°C, a continuous SiO_2_‐rich glass layer stabilized emissivity near 0.98. At higher temperatures (∼1768°C), the volatilization of SiO_2_ exposed underlying ZrO_2_, decreasing emissivity to ∼0.85. Moreover, SiO_2_ volatilized more readily in the dynamic plasma environment than in static air, highlighting the importance of testing under realistic flight conditions.

### 4.4 Microstructural Engineering for Tailored Radiative Properties

**FIGURE 12:**
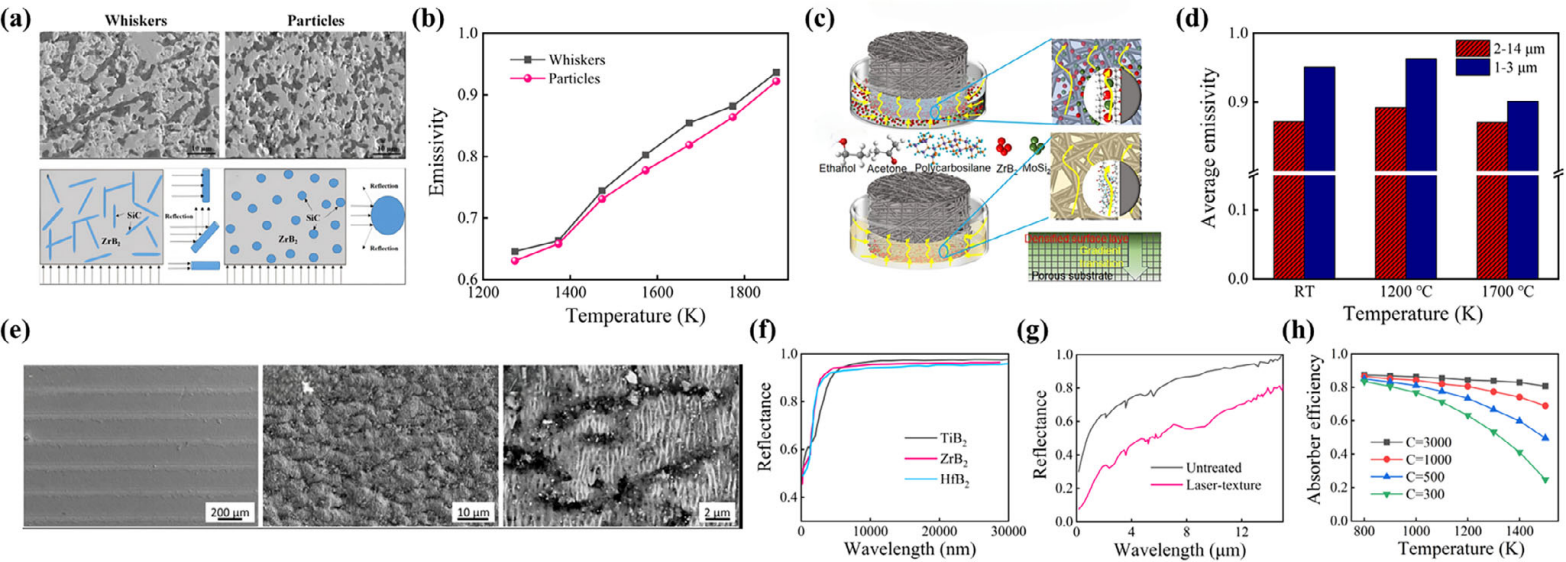
Radiation properties and microstructure of tailored ultra‐high temperature ceramics (UHTCs). (a) The characteristic microstructure of whiskers and particles modified ZrB_2_‐SiC, and the schematic diagrams of two materials showing the interaction of electromagnetic waves with a cylinder and a sphere. Reproduced with permission [52]. Copyright 2015, Elsevier. (b) Comparison of the total emissivity of whiskers and particles of modified ZrB_2_‐SiC as a function of measurement temperature [52]. (c) Fabrication of gradient dense layers. Reproduced with permission [222]. Copyright 2024, Youke Publishing. (d) Average emissivity of the ZrB_2_‐MoSi_2_‐SiC dense layer material [222]. (e) SEM images of femto‐second laser textured TaB_2_ sample (The images in the middle and right columns are referred to as groove area). Reproduced under the terms of the CC BY 4.0 license [223]. Copyright Sani et al. (f) The theoretical reflectance spectra of TiB_2_, ZrB_2_ and HfB_2_ [224]. (g) Hemispherical reflectance spectra of the untreated a laser‐textured sample [223]. (h) Calculated temperature‐dependent absorber efficiencies at different concentration ratios [223].

Microstructure, as another important factor that influences the surface radiation, offers opportunities to tailor their performance for specific applications. By engineering microstructure, it is possible to achieve not only higher emissivity in the infrared band but also improved solar spectral selectivity. As noted earlier, SiC is a common secondary reinforcing phase used to enhance the properties of the ceramics, typically introduced in particulate form. However, the morphology of the SiC phase strongly affects radiative behavior. Wang et al. [52] investigated the impact of SiC's whiskers and particles' shape on the emissive properties of the material, showing higher spectral and total emissivity for the SiC whiskers‐added sample in Figure 12b. The author noted that whisker's cylindrical geometry alters electromagnetic wave propagation by concentrating reflections along specific orientations, and the reduced surface‐area‐to‐volume ratio minimizes the interfacial reflection energy loss, allowing more efficient radiative energy transmission, as shown in Figure 12a. For both samples, the formation of a SiO_2_‐rich glassy layer during the pre‐oxidation process also promotes the emissivity enhancement. Beyond radiative performance, SiC whiskers also act as mechanical tougheners to strengthen the major ZrB_2_ phase and stabilize the SiO_2_ glass layer at high temperatures, enhancing the overall performance of ZrB_2_ UHTCs.

Inspired by plant transpiration, Yang et al. [222] developed a novel gradient‐architected as shown in Figure 12c. In the study, ceramic slurry was directed into specific zones of the fibrous structure, thus forming a three‐layer gradient design (dense–transition–substrate) with a smooth, defect‐free surface and uniform element distribution. The optimized composition (ZrB_2_:MoSi_2_ = 6:4) exhibited outstanding radiative properties, with average emissivity peaks at 1200°C at 0.963 and 0.892 within the 1–3 µm and 2–14 µm range, respectively, as shown in Figure 12d. The overall emissivity remained above 0.9 within 1–3 µm and above 0.87 within 2–14 µm range, showing excellent radiative performance. This enhancement is linked to the unique electronic structure of MoSi_2_, which combines metallic and nonmetallic properties, enabling stronger electronic transitions and absorption in the short‐wavelength infrared regime. In addition to the excellent radiative properties at high temperature up to 1700°C, the layer exhibits strong and persistent ablation resistance, with a minimum linear ablation rate of 0.012 µm·s^−1^ under 1.5 MW·m^−2^ for 1000 s, together with the enhanced ceramic‐substrate bonding and mechanical properties brought by the gradient dense layer. Collectively, these features highlight the exceptional potential of gradient ZrB_2_‐MoSi_2_‐SiC coatings for extreme‐environment applications.

Beyond doping and bulk microstructural design, surface treatments provide an effective route to tailor the spectral selectivity of UHTCs. Xiang et al. [224] analyzed the theoretical potential of TMB_2_ (TM = Ti, Zr, and Hf) as solar absorbers. The materials have typical spectrally selective reflectance with the well‐recognizable step‐like profile as shown in Figure 12f. The reflectance is low in the visible and near infrared wavelength range and is relatively high in the mid‐infrared range, which meets the fundamental demands for selective solar absorption, though further optimization is needed for practical efficiency. As reported in the research of Azzali et al. [225], surface features (porosity, roughness, texture, and so on) are important factors that affect the radiative properties of the material, and the size of surface features is the key parameter. Due to light trapping of corresponding spectral components, surfaces with a feature size similar to the peak wavelength of blackbody radiation will have a higher absorptance. Thus, researchers have also tried surface texture to enhance the radiation properties of UHTCs.

In 2023, Sani et al. [223] first reported the successful photothermal efficiency enhancement of ultra‐hard ceramics, TaB_2_, using laser machining. The Laser‐Induced Periodic Surface Structure (LIPSS) caused by laser treatment in Figure 12e increases the solar absorption efficiency by around 45% compared to the untreated sample (Figure 12g). The hierarchical micro‐grooves and ridges effectively trapped broadband solar radiation through multiple reflections. Besides, laser‐induced chemical changes, such as Ta_x_O_y_ oxidation, narrowed the electronic bandgap and further enhanced the absorption in the visible spectrum. In practical solar‐thermal systems, efficiency generally decreases with rising temperature. However, as shown in Figure 12h, the average efficiency loss from 800 to 1500 K is only 7% under a high solar concentration ratio of 3000 using the laser‐treated TaB_2_ material, making the laser‐textured ceramic absorbers suitable for high concentration ratio solar systems operating at ultra‐high temperature.

In a consequent study, Sani et al. [226] induced LIPSS on LaB_6_ by means of a femtosecond‐laser. The combined effects of oxide removal and surface structuring led to reduced IR emissivity while enhancing absorption, yielding a solar‐thermal efficiency reaching 60.6% at 1000 K under 1000× solar concentration—one of the highest reported values. In parallel, chemical etching techniques have also been employed to texture UHTCs' surfaces, achieving high efficiency under ∼3000× concentration ratio at 1000 K [227].

## 5 Optimization Strategy for Thermoelectric Properties of UHTCs: as Matrix and as Reinforcing Phase

**FIGURE 13:**
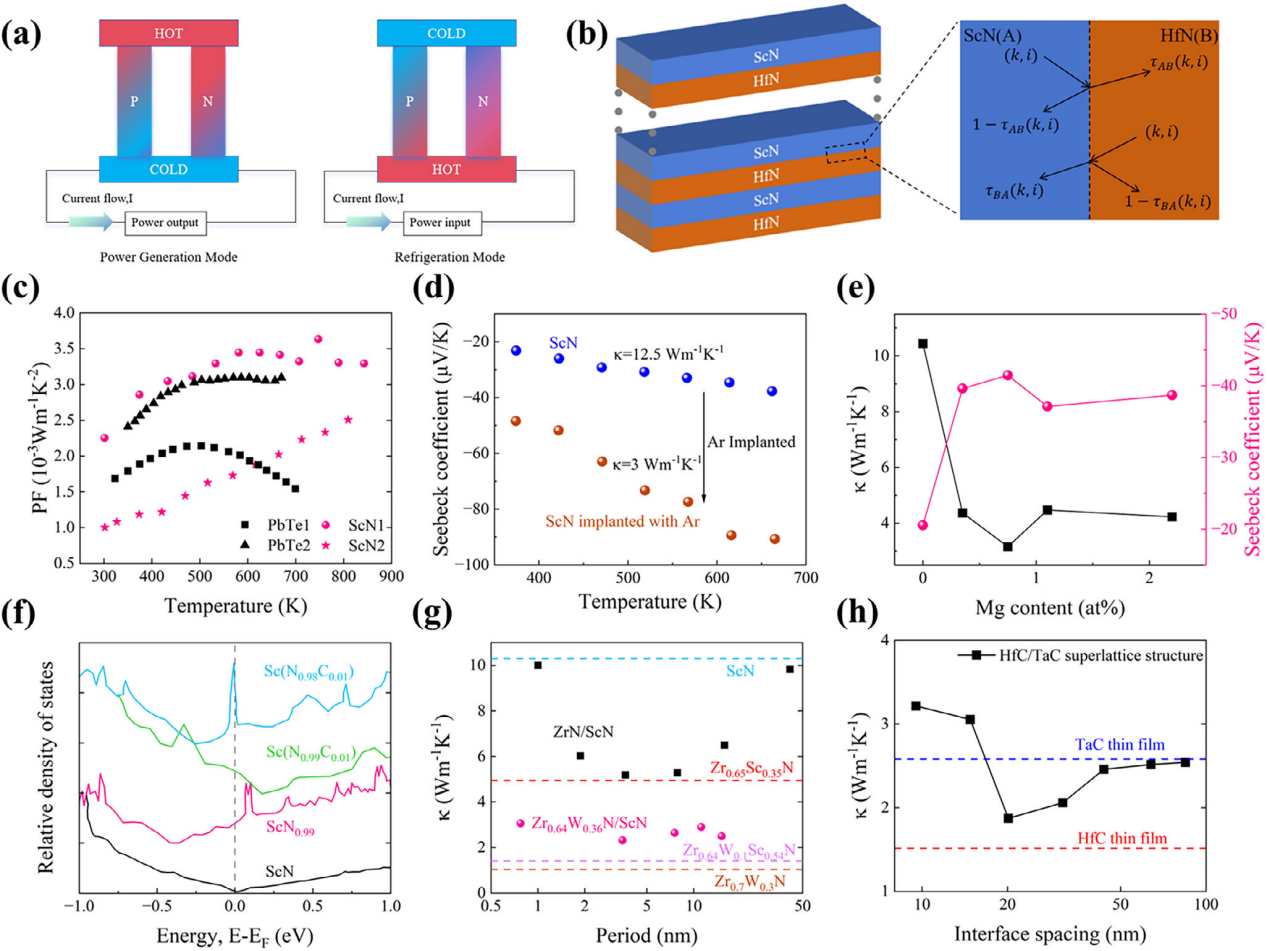
Ultra‐high temperature ceramics (UHTCs) for thermoelectric conversion. (a) Thermoelectric working principle. (b) Schematic diagram and cross‐plane thermal transport characteristics of ScN/HfN superlattice structure [235]. τ_AB_ represents the mode‐specific transmission of phonons from material A to material B [242]. k and i represent the phonon wave vector and polarization, respectively. (c) Power factor of ScN compared to PbTe [233, 234]. (d) Temperature dependence of the Seebeck coefficient of samples before (blue) and after (red) Ar implantation. After injecting Ar, the thermal conductivity at room temperature decreased from 12.5 to 3 Wm^−1^K^−1^ [239]. (e) The thermoelectric properties of Mg‐doped SCN were improved [240]. (f) Example of effects of vacancies and dopants on the band structure of ScN [241]. (g) Cross‐plane thermal conductivity of 300 nm thick ZrN/ScN (squares) and Zr_0.64_W_0.36_N/ScN (dots) multilayers [243]. Superimposed on the plot are horizontal lines corresponding to the experimentally determined lattice component of thermal conductivity, i.e., the alloy limit of different ZrN, ScN, and W_2_N alloys. (h) Room temperature cross‐plane thermal transport properties of HfC/TaC SLs as a function of SL interface spacing [244].

Thermoelectric (TE) materials can directly convert thermal energy into electrical energy. The pursuit of high‐performance thermoelectric materials capable of operating in extreme environments represents a significant frontier in materials science and energy engineering, such as waste heat recovery and deep‐space power generation. The figure of merit (ZT) is used to assess thermoelectric materials and is measured by three competing parameters, *ZT*  = *S* ^2^ *T*σ/κ, where S is the Seebeck coefficient, σ is the electrical conductivity, T is the temperature, and κ is the thermal conductivity. Its principle is shown in Figure 13a. In this technology, the temperature difference is utilized to facilitate the migration of charge carriers, thereby generating electricity. However, these parameters are often intrinsically interdependent, making the optimization of ZT a formidable challenge.

Conventional thermoelectric materials usually fail for ultra‐high temperature applications (> 1500°C) due to low melting points, chemical instability, or elemental volatility in oxidizing or corrosive environments [228, 229]. UHTCs, owing to their unique properties, provide a novel material platform for thermoelectric applications in extreme environments. As covalent/metal bond hybrid materials with melting points exceeding 3000 K, UHTCs exhibit the triple advantages of intrinsic stability, high electrical conductivity [230, 231, 232], and controllable thermal transport behavior in extreme environments. However, the intrinsic thermoelectric ZT of most UHTCs is suboptimal, primarily due to their relatively high thermal conductivity and low Seebeck coefficient. Consequently, recent research has diverged into two distinct yet complementary strategies aimed at engineering UHTCs for enhanced thermoelectric performance. The first strategy employs UHTCs as the host matrix, tailoring their inherent properties through microstructural design, including doping and compositing with secondary phases. These modifications seek to decouple the interrelated electronic and thermal transport parameters—for instance, by introducing phonon‐scattering centers to reduce lattice thermal conductivity without significantly compromising electrical conductivity. The second strategy represents a reverse approach: instead of using UHTCs as the host, it leverages them as a functional reinforcing phase. In this method, robust and stable UHTCs nanoparticles or nanostructures are incorporated into other promising thermoelectric matrices. The role of the UHTCs phase is to enhance the thermal stability of the composite, refine the microstructure, and introduce additional interfaces for selective phonon scattering, thereby improving the overall thermoelectric ZT of the base material. By examining these strategies, this section aims to provide a comprehensive overview of the pivotal role UHTCs play in advancing the frontiers of high‐temperature thermoelectrics.

### 5.1 Performance Optimization of UHTCs‐Based Composite Thermoelectric Materials

Scandium nitride (ScN) is one of the most promising thermoelectric materials among all UHTCs due to its high‐power factor and moderate bandgap control ability. Its superlattice structure has become a key technological path to overcome its thermal conductivity limitations. ScN exhibits a high thermoelectric power factor [233, 234] (*PF*  = *S* ^2^ σ), as shown in Figure 13c. Kerdsongpanya et al. [233] reported the excellent thermoelectric properties of ScN up to 800 K, including a high‐power factor of up to 2.5 × 10^3^ Wm^−1^K^−2^, a relatively high Seebeck coefficient of ‐86 µV/K, and a metal‐like electrical resistivity of 2.94 µΩ·m. It is estimated that the lower limit of its ZT value is ∼0.2 at 800 K. Burmistrova et al. [234] demonstrated epitaxial growth of ScN (001) thin films on magnesium oxide (001) substrates via the direct current reactive magnetron sputtering method. Studies have shown that within the temperature range of 600 to 840 K, the thin films exhibit remarkable thermoelectric properties: the power factor up to 3.3–3.5 × 10^3^ Wm^−1^K^−2^, the electrical resistivity as low as 6.67 µΩ·m, and a cross‐sectional thermal conductivity of 8.3 Wm^−1^K^−1^ at 800 K. The ZT value is estimated to be 0.3. ScN's high power factor is attributed to its moderate bandgap, allowing the tuning of the Schottky barrier height [47, 235, 236], which is a key parameter of the energy filtering mechanism and can be used to adjust the Seebeck coefficient and optimize the power factor. Its PF is equivalent to traditional thermoelectric material PbTe (Figure 13c) [237, 238]. Reducing thermal conductivity is an effective means to improve the thermoelectric performance of ScN. Burcea et al. [239] prepared point and extended defects in (111) ScN thin films grown by epitaxial growth using argon ion implantation technology. By increasing phonon defect scattering, the thermal conductivity at room temperature was decreased from 12.5 to 3 Wm^−1^K^−1^, and the Seebeck coefficient was increased, as shown in Figure 13d. Tureson et al. [240] improved the Seebeck coefficient by adding magnesium to ScN, resulting in a 20% increase in thermoelectric power factor by reducing thermal conductivity (Figure 13e). The calculations by Kerdsongpanya et al. [241] indicate (Figure 13f) that there are possibilities of obtaining a large Seebeck coefficient without reducing the electrical conductivity. By introducing an appropriate dopant with the presence of N vacancies, the Fermi level can be moved near the location of the vacancy peak in the DOS, which could yield an enhancement of the thermoelectric power factor. In addition, these dopants can effectively reduce the lattice thermal conductivity caused by phonon scattering at point defects.

A superlattice (SL) structure is a periodically layered thin film architecture, artificially engineered with alternating layers of two or more compositionally and functionally distinct materials in a specific thickness ratio. It can effectively suppress cross‐plane thermal conduction by enhancing phonon scattering and band engineering at heterointerfaces. Simultaneously, it modulates the Schottky barrier height to enable efficient energy filtering during electron transport, therefore improving the Seebeck coefficient while preserving high electrical conductivity, and offering significant advantages for thermoelectrics. Taking the ScN/HfN SL as an example, the schematic diagram and out‐of‐plane thermal transport characteristics of its superlattice structure are shown in Figure 13b. Saha et al. [235] conducted first principles calculations on the electronic structures, phonons, and thermal properties of ZrN, HfN, and ScN. Their results revealed closely matched lattice constants between ScN and HfN (and a minimal 1.5% mismatch between ScN and ZrN), confirming their suitability for epitaxial superlattice growth. Furthermore, the large phonon spectral gap mismatch between ZrN/HfN and ScN reduces phonon thermal conductivity. ScN/HfN SL exhibits lower predicted lattice thermal conductivity than ScN/ZrN due to HfN's wider gap between acoustic and optical phonons. Subsequent calculations [245] confirmed ScN/HfN SL achieves thermal conductivity 1–2 orders of magnitude lower than bulk materials. Zebarjadi et al. [47] utilized the metal/semiconductor interface barrier for energy filtering: only electrons surpassing this barrier contribute to transport. The screening of high‐energy carriers significantly enhanced the Seebeck coefficient to 820 µV/K at room temperature, alongside a low thermal conductivity of 1.8 Wm^−1^K^−1^. Modeling predicts a ZT of 1.5 at 1300 K based on these parameters, assuming conserved lateral electron momentum across the interface. As shown in Figure 13g, Rawat et al. [243] observed that the cross‐plane thermal conductivity of ZrN/ScN multilayers is strongly period‐dependent, reaching a minimum of 5.25 Wm^−1^K^−1^ at a 6 nm period (comparable to Zr_0.65_Sc_0.35_N alloy lattice conductivity). Incorporating WN_x_ alloying into ZrN layers to form (Zr, W)N/ScN SL further suppressed phonon transport via alloy scattering, reducing thermal conductivity below 2 Wm^−1^K^−1^ (minimum 1.8 Wm^−1^K^−1^) with minimal dependence on period thickness. Liang et al. [244] fabricated periodic HfC/TaC transition metal carbide SL with periods ranging from 9.5 to 84.5 nm (Figure 13h). Measured thermal conductivity varied with period length, achieving an ultralow value of 1.8 Wm^−1^K^−1^ at room temperature. Adding a 20 nm HfO_2_ cap layer effectively prevented oxidation of the HfC/TaC structure while maintaining low thermal conductivity, enabling novel high‐temperature insulation and hot spot management solutions.

**FIGURE 14:**
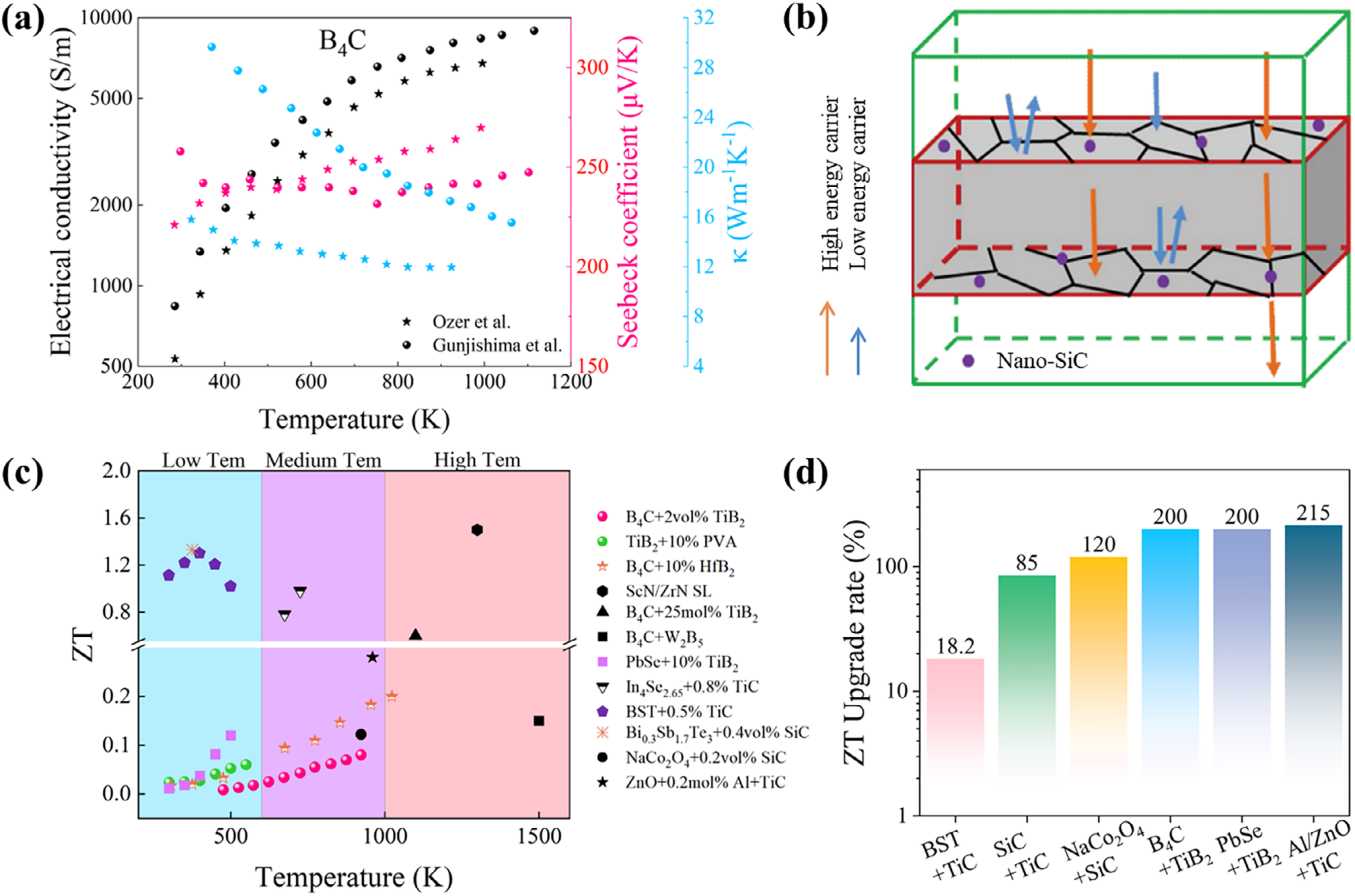
Potential of ultra‐high temperature ceramics (UHTCs) in the thermoelectric field. (a) Thermoelectric properties of B_4_C [42, 44]. (b) Schematic diagram of BiSbTe doped with nano‐SiC. Reproduced with permission [253]. Copyright 2013, Wiley. SiC nanoparticles can block low‐energy charge carriers through the energy filtering effect. (c) Potential of UHTC in the Thermoelectric Field. ZT value of typical thermoelectric materials doped with UHTC. (d) Performance improvement of thermoelectric materials after doping with UHTCs.

Boron carbide, as a typical p‐type high‐temperature semiconductor, exhibits excellent thermal stability and mechanical properties [246, 247]. As shown in Figure 14a, B_4_C demonstrates well‐balanced thermoelectric properties, positioning it as a highly promising matrix material for high‐temperature thermoelectric applications among carbide ceramics [42, 44]. Its electrical conductivity increases significantly with temperature, a characteristic of semiconducting behavior: from ∼500 to 800 S/m at room temperature to ∼9000 S/m at 1000 K. Concurrently, B_4_C maintains a relatively high Seebeck coefficient (∼250 µV/K) for effective thermoelectric potential generation. Its thermal conductivity is inherently low and further decreases with rising temperature due to intensified phonon‐phonon scattering, effectively mitigating the typical thermoelectric trade‐off between electrical and thermal conductivity. The synergistic advantages—low thermal conductivity, moderate electrical conductivity, and moderate Seebeck coefficient—establish B_4_C as a promising matrix for high‐temperature thermoelectrics. However, its relatively low ZT value limits practical application. Enhancing electrical conductivity represents the most effective route to improve thermoelectric efficiency. Gunjishima et al. [248] prepared directionally solidified B_4_C‐TiB_2_ composites using the floating zone method and reported that B_4_C + 25 mol% TiB_2_ had a ZT value of 0.6 at 1100 K. Feng et al. [249] prepared B_4_C‐TiB_2_ composite materials by in situ reactive spark plasma sintering of B_4_C with the addition of nano‐TiO_2_ powder. The study showed that 10 wt.% TiB_2_ doping increased the conductivity by 220%, while reducing the thermal conductivity by 31%, ultimately doubling the ZT value. Cai and Nan [250] prepared B_4_C‐W_2_B_5_ composite material by introducing WC phase through ball milling and hot pressing method, and obtained a ZT value of about 0.15 at 1500 K. Ozer et al. [251] further optimized and found that only 2 vol.% TiB_2_ doping is needed to increase the conductivity by 300% and the ZT value by 120%. Innocent et al. [252] prepared B_4_C/HfB_2_ composite materials by sintering a mixture of B_4_C and HfB_2_ powders using discharge plasma, and the thermoelectric properties of the 10 wt.% HfB_2_‐B_4_C composite material achieved a ZT value of 0.2 at 1000 K.

Despite inherent limitations when used individually—ScN requires further thermal conductivity reduction to improve ZT, while B_4_C suffers from low electrical conductivity—both materials demonstrate significant potential for medium‐to‐high temperature thermoelectrics due to their unique crystal structures and electronic transport properties. Current research confirms the feasibility of enhancing ScN‐based ZT values. Future efforts should focus on optimizing interface quality, precisely tuning barrier heights and alloy compositions, or implementing multi‐scale structural designs that could surpass current ZT limits. For B_4_C, incorporating highly conductive UHTC dopants or secondary phases effectively supplements carrier supply and improves transport pathways, dramatically enhancing electrical conductivity and thus improving the ZT value. Research on ScN and B_4_C‐based thermoelectrics not only advances performance modulation theory for UHTCs systems but also establishes critical design principles and technical pathways for developing efficient, stable next‐generation medium‐to‐high temperature thermoelectric materials.

### 5.2 Application of UHTCs as Reinforcing Phases in Thermoelectric Materials

The core advantage of UHTCs lies in their performance at high temperatures and high electrical conductivity, making them one of the best choices for high‐temperature thermoelectrics. However, most UHTCs exhibit very high electrical conductivity (such as ZrB_2_ with a conductivity of about 10^7^ S/m [230]), but low Seebeck coefficients [12] (about 10–30 µV/K), resulting in a limited power factor (S^2^σ). Their semi‐metallic/metallic nature leads to high carrier concentration and narrow energy distribution, constraining Seebeck coefficient improvement. Conversely, many materials possess high Seebeck coefficients but low conductivity. Consequently, UHTCs serve as effective dopants to enhance thermoelectric performance. Strategic doping can enhance carrier concentration to boost the power factor. Additionally, doping introduces defects that impede phonon transport, reducing thermal conductivity and further improving thermoelectric efficiency.

The nano composite material method has been widely studied in the field of thermoelectric technology [254, 255, 256], providing new ideas for simultaneously improving thermoelectric and mechanical properties. Li et al. [257] doped TiC nanoparticles into In_4_Se_2.65_, and the electrical resistivity and thermal conductivity decreased with the addition of TiC. The ZT value at 750 K was 0.98. Utilizing the dispersion strengthening effect of nano inclusions, the mechanical properties have been effectively improved. The addition of 0.8 wt.% TiC resulted in a flexural strength of 73 MPa, which is 40% higher than the original sample. Zhao et al.’s study [258] also reached a similar conclusion, that 0.5 wt.% TiC increased the ZT value of Bi_0.3_Sb_1.7_Te_3_ (BST) to 1.3 at 400 K, which is 18.2% higher than pure BST (ZT = 1.1 at 400 K), and the hardness increased by 38%. Li et al. [253] achieved the synergistic enhancement of thermoelectric and mechanical properties by incorporating SiC nanoparticles into the p‐type Bi_0.3_Sb_1.7_Te_3_ matrix via the mechanical alloying‐spark plasma sintering method. In terms of thermoelectric properties, the sample with 0.4 vol.% SiC exhibited the optimal performance: the coherent interfaces formed between SiC and the matrix could filter low‐energy carriers through the energy filtering effect (Figure 14b), simultaneously increasing the Seebeck coefficient (reaching 211 µV/K at 427 K) and electrical conductivity (reaching 1.02 × 10^5^ S/m at 323 K), leading to a maximum power factor of 3.92 mWm^−1^K^−2^. And SiC nanoparticles enhanced phonon scattering, optimizing the lattice thermal conductivity while maintaining a total thermal conductivity similar to that of the pure matrix. Eventually, the ZT value reached 1.33 at 373 K (ZT > 1.3 in the range of 373–423 K), significantly outperforming pure Bi_0.3_Sb_1.7_Te_3_. Additionally, the high hardness and high elastic modulus of SiC reduced indentation cracks in the material, improved fracture toughness and microhardness, enhanced the machinability and device reliability of the material, and provided performance guarantees for thermoelectric device fabrication. Zhang et al. [259] achieved efficient enhancement of the thermoelectric properties of NaCo_2_O_4_ by introducing nano‐SiC into its matrix via the solid‐state method. Nano‐SiC is dispersed in the grain boundaries of NaCo_2_O_4_, inhibiting grain growth and thereby forming a large number of new grain boundaries and interfaces, which enhances phonon scattering and significantly reduces thermal conductivity. Simultaneously, by increasing electrical conductivity (71 S/cm for 0.4 vol.% SiC samples) and Seebeck coefficient (227 µV/K for 1.0 vol.% SiC samples), a maximum power factor increases of 70% is achieved compared to pure NaCo_2_O_4_. The ZT value of the final 0.2 vol.% SiC sample reached 0.122 at 650°C, which is approximately twice that of pure NaCo_2_O_4_ (0.061). Manyedi et al. [260] prepared Al‐doped ZnO (Al‐ZnO) and 2D TiC‐modified Al‐ZnO nanocomposites via the hydrothermal method and mechanical alloying. Al doping improved the electrical conductivity by increasing the carrier concentration, while TiC nanosheets formed heterogeneous interfaces with Al‐ZnO. These interfaces screened low‐energy carriers through the energy filtering effect, resulting in a Seebeck coefficient of ‐105 µV/K at 960 K. Furthermore, the multi‐scale grain boundaries and quantum well structures introduced by TiC enhanced phonon scattering, reducing the thermal conductivity of ZnO from 7.62 to 5.75 Wm^−1^K^−1^ at 1000 K. Eventually, the 2 mol% Al‐ZnO‐TiC composites achieved a ZT value of 0.28 at 960 K, which was a significant improvement compared to pure Al‐ZnO. In addition, the high stability of TiC increased the thermal decomposition temperature of the material, providing support for the material's mechanical stability and device processability. Gall et al. [261] reported the electrical properties of Sc_1‐x_Ti_x_N, and found that when x = 0.31, the conductivity increased by 1200% compared to ScN. Malik et al. [12] synthesized TiB_2_ via the sol–gel and carbothermal reduction method, and optimized its thermoelectric performance through two strategies: “binder regulation” and “heterogeneous compositing”. On one hand, polyvinyl alcohol (PVA, 3–10 wt.%) was introduced into TiB_2_. The residual carbon generated by PVA pyrolysis and the formed pores significantly reduced the thermal conductivity of TiB_2_ by 95% (from 25 to 0.2 Wm^−1^K^−1^ at 550 K) through multi‐scale phonon scattering. Meanwhile, the sample doped with 10 wt.% PVA exhibited an electrical conductivity of 2.5 × 10^4^ S/m at 300 K, and a Seebeck coefficient of 36.3 µV/K at 550 K, with the ZT value increased to 0.064. On the other hand, 10 wt.% TiB_2_ was incorporated into PbSe to form a composite material. The high electrical conductivity of TiB_2_ enhanced the electrical conductivity of PbSe by 9 times (reaching 2.8 × 10^3^ S/m at 400 K) while retaining the intrinsic high Seebeck coefficient of PbSe (‐342 µV/K at 550 K). Eventually, a power factor of 280.2 µWm^−1^K^−2^ was achieved at 495 K, and the ZT value was increased to 0.12, which is significantly superior to that of pure PbSe and traditional TiB_2_‐based composites.

The utilization of UHTC as a reinforcing phase or dopant presents a highly promising and versatile strategy for advancing thermoelectric materials. Beyond the primary goal of enhancing the thermoelectric figure of merit (Figure 14c,d) through mechanisms such as phonon scattering at interfaces and energy filtering effects, the incorporation of UHTCs concurrently bestows exceptional mechanical robustness, thermal stability, and creep resistance upon the composite system.

Notably, UHTCs also show significant potential in high‐temperature thermal sensing [262, 263, 264, 265, 266]. The stable thermoelectric response above 1500 K—coupled with oxidation/corrosion resistance—enables precise temperature monitoring in extreme applications like aeroengine exhausts and industrial furnaces. Additionally, their excellent compatibility with metal electrodes allows their use as compensating electrodes or novel thermoelectric arms in high‐temperature thermocouples. The use of UHTCs overcomes limitations of traditional noble‐metal thermocouples (e.g., high‐temperature softening, short service life), expanding measurement temperature limits and stability. Future refinements in UHTCs composition design, interface control, and composite processing will not only advance thermoelectrics toward higher ZT values and improved mechanics but also bridge the gap from potential to practical deployment in specialized sensing fields.

## 6 Summary and Outlook

In summary, we conduct a systematic review and in‐depth discussion on the complete research chain of UHTCs, covering fundamental physical mechanisms, cutting‐edge discovery methods, and applications in extreme environments. By reviewing the research progress in key fields such as high‐temperature thermal transport physics, regulation of thermal/radiation/ thermoelectric properties of materials, it comprehensively presents the development context and future application potential of this field.

First, starting from the core mechanism of high‐temperature thermal transport: the thermal transport process is essentially a complex system co‐dominated by phonons, electrons, and photons, with mutual coupling among the three. The traditional phonon‐dominated model is no longer sufficient to accurately describe the thermal behavior under extreme conditions; the roles of electron‐phonon coupling and intrinsic radiative thermal transfer become increasingly critical. This insight lays a theoretical foundation for the accurate prediction and regulation of the thermophysical properties of UHTCs. On this basis, we systematically review the thermal conductivity characteristics of four major categories of UHTCs (oxides, borides, carbides, and nitrides). By comparing theoretical calculations with experimental data, it profoundly reveals the intrinsic structure‐property relationships between crystal structure, chemical bond characteristics, and phonon/electron transport capabilities. Meanwhile, it proposes multi‐dimensional thermal regulation strategies, providing feasible technical pathways for optimizing the high‐temperature thermal stability of materials. Further, we emphasize the key role of the radiation properties of UHTCs in thermal protection and energy conversion scenarios: the intrinsic properties of materials, doping, surface oxidation, and microstructure treatment can all significantly alter their spectral radiation behavior. Achieving the “on‐demand design” of the radiation properties of UHTCs is precisely the core breakthrough for developing next‐generation intelligent thermal management systems and high‐efficiency radiative cooling technologies. Finally, we extend its research perspective to the thermoelectric application of UHTCs: through regulatory approaches such as band engineering, microstructure optimization, and introduction of scattering centers, the thermoelectric ZT of UHTCs can be effectively improved while ensuring their excellent high‐temperature stability. This provides a new solution for high‐temperature thermoelectric power generation scenarios.

Although current research on UHTCs has made progress in multiple dimensions, there remain various challenges and room for breakthroughs to meet the demands of extreme application scenarios. Future efforts should focus on the following directions:

### 6.1 In‐Depth Exploration of Fundamental Physical Mechanisms

There are still obvious limitations in the current understanding of the thermal transport mechanisms of UHTCs under extremely high temperatures. On one hand, it is necessary to further investigate the dynamic evolution law of electron‐phonon coupling in high‐temperature and high‐pressure environments, and clarify the intrinsic competitive mechanism between radiative heat transfer and phonon/electron transport under extreme conditions. On the other hand, it is essential to establish a theoretical model for the thermal behavior of UHTCs under multi‐physical fields (synergistic effects of heat, force, and radiation) to make up for the applicability defects of existing theories in complex environments and provide theoretical support for the accurate prediction of material thermal behavior in extreme scenarios. Taking ZrB_2_ as an example, although extensive experimental thermal conductivity data are available for temperatures as high as 2273 K, accurate computational assessments of the electronic contribution remain insufficient. To address this, first‐principles calculations based on Density Functional Theory and Density Functional Perturbation Theory can be performed to characterize the electronic structures, phonon spectra, and electron‐phonon interactions.

### 6.2 Precise Regulation of Crystal Quality and Low‐Cost Synthesis

Existing UHTCs are confronted with the core dilemma of scarce single crystals and high synthesis costs, which severely restrict the exertion of their intrinsic properties and engineering applications. On one hand, the preparation of high‐quality UHTCs single crystals relies on complex processes such as the high‐temperature melt method and the vapor transport method. These processes not only require breaking through extreme temperature conditions but also suffer from problems such as slow crystal growth rate and high defect density, leading to high preparation costs of single crystals and thus making large‐scale application difficult. On the other hand, during the sintering process of polycrystalline UHTCs, abnormal grain growth and excessive porosity are prone to occur, causing significant fluctuations in thermal and mechanical properties, which cannot stably meet the requirements of extreme scenarios for material performance consistency. Future efforts need to achieve synergistic breakthroughs from the dual dimensions of synthesis technology innovation and cost control: First, develop new low‐cost pathways for single crystal preparation, such as optimizing the flux system to reduce the single crystal growth temperature, or improving directional solidification technology to realize rapid single crystal growth, while accurately controlling the crystal defect density through in situ defect monitoring technology. Second, for polycrystalline materials, explore the “low‐temperature sintering‐densification synergy” process to achieve low porosity while reducing the sintering temperature and simultaneously improve material uniformity. Third, promote the large‐scale adaptation of synthesis processes, such as developing continuous sintering equipment to replace batch equipment, reducing mass production costs, and laying a foundation for the transformation of UHTCs from laboratory samples to engineering components.

### 6.3 Development of Precise Thermal Conductivity/Emissivity Characterization Technology Under Extreme Environments

Current characterization methods are unable to accurately measure the thermal conductivity or emissivity of UHTCs under extreme environments (e.g., 3000 K high temperature, high pressure, oxidizing/corrosive atmospheres), resulting in significant deviations between measured data and the actual service performance of materials. Conventional thermal conductivity testing methods (e.g., the laser flash method) are mostly limited to inert environments below 2000 K, which cannot capture the dynamic regulatory effect of material phase transformation and microstructure evolution on thermal conductivity under extremely high temperatures. Meanwhile, there is no mature characterization method for thermal conductivity measurement under high pressure or oxidizing/corrosive atmospheres, leading to a lack of accurate experimental support for the prediction of material thermal behavior in extreme scenarios. Also, for the emissivity measurement, many test methods are limited to below 2000 K due to the degradation of the thermal stability of the heating elements and the transmittance of the optical windows. Besides, considering the detector response constraints, only a certain wavelength range can be measured in certain experiment setups. Methods like two‐color pyrometry, relying heavily on the gray body assumption, still have major uncertainties and cannot be used as a standard reference yet. Therefore, developing precise thermal characterization technology adapted to extreme environments has important research value and application significance. Implementing contactless heating platforms—such as laser heating, electromagnetic levitation, or concentrated solar furnaces—will enable stable and controllable measurements from ∼2000 to ∼3000 K. Broadband spectroscopic tools like Fourier‐transform infrared spectrometers can also be applied to extend the spectral range for continuous, multi‐spectral data acquisition. Meanwhile, advances in high‐pressure measurement techniques will provide critical experimental support for a deeper understanding of the intrinsic heat transport mechanisms of UHTCs under extreme conditions. In situ high‐pressure measurement based on the diamond anvil cell is currently the most mature and widely used technical approach, with its core principle being the application of pressures reaching hundreds of gigapascals or even higher to sample amounts while simultaneously conducting property measurements using various in situ characterization techniques.

### 6.4 Synergistic Innovation of Performance Regulation Strategies

Existing thermal regulation, thermoelectric optimization, and radiation management strategies for UHTCs are mostly limited to single‐dimensional design, which cannot meet the demand for multi‐functional integration of materials in extreme scenarios. Future research should promote multi‐mechanism synergistic regulation, for example, combining heterogeneous element doping with microstructure design to achieve the simultaneous optimization of thermal conductivity, thermoelectric ZT, and spectral radiation properties; or purse the targeted radiative value as well as the anti‐ablation performance. At the same time, explore the cross‐scale integrated regulation scheme of “heat conduction‐thermal radiation‐thermoelectric conversion” to break through the limitation of a single function and realize the multi‐functional integration and efficient service of materials in extreme environments.

### 6.5 Dynamic and Post‐Test Experiments for Long‐Term and Real Environment Application

Some research in a plasma wind tunnel has revealed that the performance of the UHTCs in the dynamic environment, like surface catalyticity, spectral emissivity evolution, and oxide layer morphology, differs a lot from that in the static air environment. However, present studies mainly concentrate on static environment cases or the radiative and ablation properties change with temperature, oxidation, and so on. Besides, few studies report the radiative or other performance after the temperature‐ramped oxidation experiments, which is critical for evaluating long‐term applicability and durability. Thus, future research should prioritize dynamic testing environments and time‐resolved diagnostics to bridge the gap between laboratory oxidation studies and operational performance in hypersonic or aerospace scenarios. First, moving beyond static testing by implementing plasma wind tunnels, inductively coupled plasma facilities, and oxyacetylene torch systems can reproduce the coupled aerothermal‐chemical process of hypersonic flight. Second, deploying in situ and time‐resolved diagnostics can capture the dynamic evolution of UHTC surfaces during oxidation or ablation, helping to reveal the deep mechanisms. Promising approaches include laser‐induced fluorescence, laser thermogravimetric analysis, high‐speed pyrometry, and so on. Third, quantifying the effects of oxide layer thickness, phase transformations, thermal conductivity, and spectral emissivity of the materials tested after controlled thermal excursions that simulate real‐world duty cycles is needed for better lifetime prediction models.

## Conflicts of Interest

The authors declare no conflicts of interest.

## Data Availabilty Statement

The authors have nothing to report.
